# Functional Selectivity and Antinociceptive Effects of a Novel KOPr Agonist

**DOI:** 10.3389/fphar.2020.00188

**Published:** 2020-03-05

**Authors:** Andrea Bedini, Lorenzo Di Cesare Mannelli, Laura Micheli, Monica Baiula, Gabriela Vaca, Rossella De Marco, Luca Gentilucci, Carla Ghelardini, Santi Spampinato

**Affiliations:** ^1^ Department of Pharmacy and Biotechnology, University of Bologna, Bologna, Italy; ^2^ Department of Neuroscience, Psychology, Drug and Children Health (NEUROFARBA), University of Florence, Florence, Italy; ^3^ Department of Chemistry “G. Ciamician”, University of Bologna, Bologna, Italy; ^4^ Department of Agricultural, Food, Enviromental and Animal Science (Di4A), Udine, Italy

**Keywords:** kappa opioid receptor, functional selectivity, intracellular signaling, chemotherapy-induced neuropathic pain, antinociception

## Abstract

Kappa opioid receptor (KOPr) agonists represent alternative analgesics for their low abuse potential, although relevant adverse effects have limited their clinical use. Functionally selective KOPr agonists may activate, in a pathway-specific manner, G protein-mediated signaling, that produces antinociception, over β-arrestin 2-dependent induction of p38MAPK, which preferentially contributes to adverse effects. Thus, functionally selective KOPr agonists biased toward G protein-coupled intracellular signaling over β-arrestin-2-mediated pathways may be considered candidate therapeutics possibly devoid of many of the typical adverse effects elicited by classic KOPr agonists. Nonetheless, the potential utility of functionally selective agonists at opioid receptors is still highly debated; therefore, further studies are necessary to fully understand whether it will be possible to develop more effective and safer analgesics by exploiting functional selectivity at KOPr. In the present study we investigated *in vitro* functional selectivity and *in vivo* antinociceptive effects of LOR17, a novel KOPr selective peptidic agonist that we synthesized. LOR17-mediated effects on adenylyl cyclase inhibition, ERK1/2, p38MAPK phosphorylation, and astrocyte cell proliferation were studied in HEK-293 cells expressing hKOPr, U87-MG glioblastoma cells, and primary human astrocytes; biased agonism was investigated *via* cAMP ELISA and β-arrestin 2 recruitment assays. Antinociception and antihypersensitivity were assessed in mice *via* warm-water tail-withdrawal test, intraperitoneal acid-induced writhing, and a model of oxaliplatin-induced neuropathic cold hypersensitivity. Effects of LOR17 on locomotor activity, exploratory activity, and forced-swim behavior were also assayed. We found that LOR17 is a selective, G protein biased KOPr agonist that inhibits adenylyl cyclase and activates early-phase ERK1/2 phosphorylation. Conversely to classic KOPr agonists as U50,488, LOR17 neither induces p38MAPK phosphorylation nor increases KOPr-dependent, p38MAPK-mediated cell proliferation in astrocytes. Moreover, LOR17 counteracts, in a concentration-dependent manner, U50,488-induced p38MAPK phosphorylation and astrocyte cell proliferation. Both U50,488 and LOR17 display potent antinociception in models of acute nociception, whereas LOR17 counteracts oxaliplatin-induced thermal hypersensitivity better than U50,488, and it is effective after single or repeated s.c. administration. LOR17 administered at a dose that fully alleviated oxaliplatin-induced thermal hypersensitivity did not alter motor coordination, locomotor and exploratory activities nor induced pro-depressant-like behavior. LOR17, therefore, may emerge as a novel KOPr agonist displaying functional selectivity toward G protein signaling and eliciting antinociceptive/antihypersensitivity effects in different animal models, including oxaliplatin-induced neuropathy.

## Introduction

Mu opioid receptor (MOPr) agonists, such as morphine, oxycodone and fentanyl, are still among the most potent and widely used analgesics; nonetheless, their high addictive potential and relevant adverse effects may significantly limit their clinical utility, thus highlighting the need for more effective and safer pain killers (Yekkirala et al., 2017). Kappa opioid receptor (KOPr) agonists have been intensively investigated in the last decade as alternatives to MOPr analgesics due to their low abuse potential and minimal adverse effects on gastrointestinal transit and respiration (Lemos and Chavkin, 2011). KOPr activation by agonists induces antinociception and may also prevent hyperalgesia produced by chronic use of MOPr targeting therapeutics (Kivell and Prisinzano, 2010; Vanderah, 2010). However, the clinical use of currently available KOPr agonists is limited by their relevant side effects, such as severe dysphoria, neuropathy-induced astrocyte proliferation and subsequent hyperalgesia, sedation, coordination impairment, and anhedonia (Bruchas and Chavkin, 2010; Bruchas and Roth, 2016). Indeed, no KOPr agonist is presently used to treat pain in humans (Bedini and Spampinato, 2018).

Functional selectivity (also called ligand-directed signaling or biased agonism) - namely, the ability of a ligand at a GPCR to selectively activate specific cell signaling pathways over others - represents an intriguing opportunity in pharmacological research, as some transduction pathways may be related to therapeutic responses, whereas others may be connected to adverse effects of a drug (Urban et al., 2007; Kenakin and Christopoulos, 2013).

In this regard, an accumulating body of evidence suggests that KOPr agonists produce antinociception and anti-itch effects *via* G protein-mediated signaling (Bruchas and Chavkin, 2010; Morgenweck et al., 2015) while their β-arrestin-2-dependent induction of p38MAPK leads to many of the relevant adverse consequences, including centrally mediated dysphoria (Bruchas et al., 2007; Ehrich et al., 2015), potassium channel heterologous desensitization (Clayton et al., 2009), neuropathy-induced astrocyte proliferation and related hyperalgesia (Xu et al., 2007), sedation, and motor incoordination (Dunn et al., 2018).

Thus, functionally selective KOPr agonists biased toward G protein-coupled intracellular signaling over β-arrestin-2-mediated pathways may be considered candidate therapeutics possibly devoid of the above-described adverse effects (Bruchas and Roth, 2016).

As regard functional selective opioid ligands it is important to remind that in October 2018, the US FDA did not approve the G-protein biased MOPr agonist oliceridine, as it was found not safer than morphine (Azzam et al., 2019); nonetheless, great interest in developing biased ligands at other GPCRs as KOPr still remains (Conibear and Kelly, 2019), and future studies analyzing novel compounds and correlating the degree of signaling bias to specific pharmacological responses are considered greatly beneficial for the whole kappa field (Mores et al., 2019).

Within this frame, by synthesizing and characterizing a series of peptide hybrids of the cyclic tetrapeptide c[Phe-D-Pro-Phe-Trp] (CJ-15,208) and the cyclic pentapeptide c[Tyr-D-Pro- D-Trp-Phe- Gly] (Cyclo EM-1) (De Marco et al., 2016), we identified a new KOPr ligand, LOR17 (c[Phe-Gly-(β-Ala)-D-Trp]); here, we describe its synthesis as well as *in vitro* and *in vivo* pharmacological characterization, with the purpose of ascertaining whether LOR17 may act as a functionally selective KOPr agonist: to do so, we investigated LOR17-mediated modulation of G protein- or β-arrestin-2-, p38MAPK-dependent signaling pathways and related cellular responses *in vitro*, as well as its *in vivo* activity in mouse models of nociceptive and neuropathic pain, by comparing its effects to those elicited by the classic KOPr reference agonist U50,488. Accordingly, in addition to determining LOR17 affinity and selectivity to KOPr, we employed different cell models to study LOR17-mediated effects on adenylyl cyclase inhibition (a G protein-dependent event), ERK1/2 and p38MAPK phosphorylation, and astrocyte cell proliferation (a p38MAPK-dependent event). With regard to this latter cellular response, it is relevant to be reminded that in different animal models of neuropathic pain endogenous dynorphin is released and promotes KOPr-dependent, β-arrestin 2-, and p38MAPK-mediated astrocytes activation and proliferation; thus leading to allodynia and hyperalgesia (Jolivalt et al., 2006; Xu et al., 2007; Aita et al., 2010). Given that also drug efficacy, and not only signaling bias, could determine differential pharmacological profiles (Conibear and Kelly, 2019), we investigated LOR17 ability to counteract U50,488-mediated increase in p38MAPK phosphorylation and the subsequent astrocytes proliferation.

cAMP ELISA and β-arrestin-2 recruitment assay were employed to characterize biased agonism; moreover, ligands’ ability to recruit β-arrestin-2 at KOPr was assessed also by means of a BRET-based assay carried out in HEK-293 cells. U50,488 was also tested in all the experimental settings listed above.

Furthermore, we compared LOR17 and U50,488 efficacy in producing antinociception in the warm-water tail-withdrawal test and in the writhing test in mice, as well as their effectiveness in counteracting thermal hypersensitivity in a mouse model of oxaliplatin-induced neuropathic pain. Finally, we assessed the effects elicited by LOR17 on motor coordination, locomotor and exploratory activities, and pro-depressant-like behavior.

## Materials and Methods

### General Procedure for the Synthesis of Cyclic Peptides

Standard chemicals were obtained from Sigma Aldrich. The synthesis was performed using a MicroSYNTH microwave lab station equipped with a built-in ATC-FO advanced fiber optic automatic temperature control. Purities were determined to be >95% by analytical reversed-phase (RP)-HPLC, performed on an Agilent 1100 series apparatus, using an RP column Phenomenex mod. Gemini 3μ C18 110 Å 100×3×3.0 mm (P/No 00D-4439-Y0); stationary phase: octadecyl carbon chain-bonded silica (C18) with TMS end-capping, fully porous organo-silica solid support, particle size 3 μm, pore size 110 Å, length 100 mm, internal diameter 3 mm; DAD 210 nm; mobile phase: from 9:1 H2O/CH3CN to 2:8 H2O/CH3CN in 20 min, flow rate 1.0 ml min, then 10 min at the same composition. Semi-preparative RP-HPLC was performed on an Agilent 1100 series apparatus using an RP column ZORBAX mod. Eclipse XDBC18 PrepHT cartridge 21.2×3×150 mm 7μ (P/No 977150-102); stationary phase: octadecyl carbon chain-bonded silica (C18), double end-capped, particle size 7 µm, pore size 80 Å, length 150 mm, internal diameter 21.2 mm; DAD 210 nm; mobile phase: from 8:2 H2O-CH3CN to 100% CH3CN in 10 min, at a flow rate of 12 ml min. ESI analysis was performed using an MS single quadrupole HP 1100MSD detector with a drying gas flow of 12.5 L/min, nebulizer pressure 30 psgi, drying gas temp. 350°C, capillary voltage 4,500 (1) and 4,000 (2), and scan range 50–2,600 amu.

### Synthesis of LOR17

The linear peptide was assembled by MW-assisted SPPS on a commercially available Wang resin preloaded with Fmoc-Phe using Fmoc-protected amino acids and TBTU/HOBt/DIPEA as coupling agents. All steps were performed according to the following general procedures.

Fmoc deprotection: The Fmoc-Phe-Wang resin (0.5 g, Phe loading 0.4–0.8 mmol/g) was treated with 20% piperidine in DMF (5 ml) for 2 min under MW irradiation at 40 W, maintaining the internal temperature at 45°C. The resin was filtered and washed with DCM (5 ml), and the treatment with 20% piperidine in DMF was repeated as described above. The suspension was then filtered, and the resin was washed three times in sequence with DCM (5 ml), MeOH (5 ml), and DMF (5 ml).

Peptide bond formation: The resin was swollen in DCM (5 ml), and a mixture of Fmoc-amino acid (0.6 mmol) and the coupling reagents TBTU/HOBt/DIPEA were added under a nitrogen atmosphere to the resin. The suspension was heated while bubbling N2 by MW irradiation. After 10 min, the resin was washed 3 times with DMF (5 ml) and MeOH (5 ml), and coupling efficacy was determined by the Kaiser test. The subsequent deprotection steps were performed as reported above.

Peptide cleavage: The resin was treated with a mixture of TFA and TIPS/water/PhOH as scavengers (7:1:1:1 v/v, 15 ml) for 2 h at r.t. After this time, the resin was filtered, and it was washed 3 times with 5% TFA in Et2O (10 ml). The filtrate and the washes were collected and removed at r.t. under N2 flow. The oily residue was suspended in ice-cold Et2O, and the precipitate was collected by centrifugation. The purity of the product was determined to be approximately 85% by RP HPLC (see General Procedure)

Cyclization: The linear peptide (0.1 mmol) in DMF (5 ml) was added over 12 h using a KD Scientific single infusion syringe pump and a 10 ml syringe to a stirred solution of HATU (0.4 mmol) and DIPEA (1.0 mmol) in DMF (20 ml). The reaction was stirred for an additional 12 h. The solvent was distilled under high vacuum, and the residue was dissolved in EtOAc (40 ml) and washed twice with 1 N citric acid (5 ml), saturated bicarbonate (5 ml) and brine (5 ml). The organic layer was dried over Na2SO4, and the solvent was removed under reduced pressure. The resulting crude residues were purified by semi-preparative RP HPLC (see General Procedure), and the cyclic peptides were characterized by reversed-phase HPLC, electrospray ionization mass spectrometry, and 1H and 13C NMR. Purities were determined to be >95% by analytical RP-HPLC and elemental analysis (see General Procedure).

1H NMR β c[Phe-Gly-β-Ala-D-Trp]1H NMR (400 MHz, [D6]DMSO) δ: 2.12 (m, 1H, β-AlaHα), 2.32 (m, 1H, β-AlaHα), 2.78 (dd, J = 5.2, 13.7 Hz, 1H, PheHβ), 2.82 (dd, J = 6.8, 14.4 Hz, 1H, D-TrpHβ), 3.02 (dd, J = 6.6, 13.7 Hz, 1H, PheHβ), 3.10 (dd, J = 8.0, 14.4 Hz, 1H, D-TrpHβ), 3.18 (m, 1H, β-AlaHβ), 3.30 (dd, J = 4.4, 15.0 Hz, 1H, GlyHα), 3.46 (m, 1H, β-AlaHβ), 3.90 (dd, J = 6.4, 15.0 Hz, 1H, GlyHα), 4.51 (ddd, J = 5.2, 6.6, 8.8 Hz, 1H, PheHα), 4.65 (ddd, J = 6.8, 8.0, 8.4 Hz, 1H, D-TrpHα), 6.71 (br.t, 1H, β-AlaNH), 6.90-6.98 (m, 2H, D-TrpArH2,5), 7.05 (dd, J = 6.8, 7.6 Hz, 1H, D-TrpArH6), 7.10-7.18 (m, 3H, PheArH2,4,6), 7.19-7.22 (m, 2H, PheArH3,5), 7.32 (d, J = 7.6 Hz, 1H, D-TrpArH7), 7.49 (d, J = 7.6 Hz, 1H, D-TrpArH4), 8.00 (br.t, 1H, GlyNH), 8.31 (d, J = 8.8 Hz, 1H, PheNH), 8.50 (d, J = 8.4 Hz, 1H, D-TrpNH), 10.70 (s, 1H, D-TrpArH1). 13C NMR (100 MHz, [D6]DMSO) δ: 26.5, 36.3, 36.6, 40.1, 44.6, 54.1, 55.2, 111.2, 112.5, 119.3, 119.4, 122.1, 124.1, 127.4, 128.3, 129.3, 137.3, 170.2, 172.0, 172.1, 172.7. ES-MS m/z [M+1] found: 462.2, calculated: 462.5. Elementary analysis for C25H27N5O4 calculated: C 65.06, H 5.90, N 15.17, found: C 65.26, H 6.00, N 15.01

### Cell Culture

HEK-293 cells (ATCC, Rockville, MD, USA, passages 25-35) stably expressing hMOPr (HEK-293/hMOPr), human delta opioid receptor (hDOPr) (HEK-293/hDOPr), or hKOPr (HEK-293/hKOPr) were obtained as previously reported (De Marco et al., 2012). HEK-293/hMOPr, HEK-293/hDOPr, HEK-293/hKOPr, and U87-MG human astrocytoma cells (ATCC, passages 3–15) were grown as monolayers in minimal essential medium (Lonza Group Ltd, Basel, Switzerland) supplemented with 2 mM L-Glutamine (Lonza), 1x non-essential amino acids (Life Technologies, Monza, Italy) and 1x antibiotic-antimycotic solution (Life Technologies), containing 10% fetal bovine serum (Life Technologies), and cultured at 37°C in a humidified atmosphere of 5% CO2.

Primary cultures of human astrocytes were obtained from Lonza (CloneticsTM Astrocyte Cell Systems, Lonza, Walkersville, MD USA), grown as a monolayer in astrocyte basal medium (ABM) supplemented with 2 mM L-Glutamine, 0.1% rhEGF, 0.25% insulin, 0.1% ascorbic acid, 0.1% GA-1000, 3% FBS and cultured at 37°C in a humidified atmosphere of 5% CO2. Human astrocytes were used in the range of passages 1–9.

### Radioligand Binding Assay

To determine KOPr protein expression levels in HEK-293/hKOR, U87-MG and normal human astrocytes, saturation binding assays were performed as follows: cell membranes were prepared as previously described (Bedini et al., 2017), and protein concentration was determined by the BCA assay kit (Thermo Fisher). For saturation binding experiments, cell membranes (40 μg) were incubated in 100 mM Tris–HCl, pH 7.4, containing 0.3% bovine serum albumin with increasing concentrations of [3H]U69,593 (20 pM - 5 nM) (PerkinElmer, Milan, Italy). Non-specific binding was determined in the presence of U50,488 (10 μM). After 90 min incubation at 25°C, bound ligand was isolated by rapid filtration on Whatman GF/C filters (Schleicher & Schuell, Dassel, Germany). Filters were washed with 20 ml of ice-cold 50 mM Tris–HCl buffer, pH 7.4, and left in scintillation fluid overnight before counting. GraphPad Prism software (GraphPad Software Inc., San Diego, CA, USA) was used to calculate receptor density (Bmax) and dissociation equilibrium constant (Kd). Data are expressed as fmol of [^3^H]U69,593 bound and normalized to cell homogenate protein content.

To evaluate affinity of LOR17 to the opioid receptors, displacement binding assays were performed in HEK-293/hMOPr, HEK-293/hDOPr, and HEK-293/hKOPr using [^3^H]DAMGO, [^3^H]diprenorphine, or [^3^H]U69,593 as MOPr-, DOPr- and KOPr-specific radioligands, respectively. DAMGO, DPDPE, and U50,488 were employed as reference MOPr, DOPr, and KOPr ligands, respectively. Cell membranes from MOPr-, DOPr-, and KOPr-expressing HEK-293 cells were prepared as previously reported (De Marco et al., 2016). Membrane preparations were incubated with 2.5 nM [3H]DAMGO, 2 nM [^3^H]-diprenorphine, or 1 nM [^3^H]U69,593 at 25°C for 90 min in buffer containing 100 mM Tris-HCl and 0.3% BSA in the presence or absence of LOR17 at various concentrations (10-12-10-4 M). Triplicate determinations were made for each experiment. Reactions were terminated by filtration through Whatman GF/C filters presoaked with 0.3% polyethylenimine, which were washed three times with 5 ml of ice-cold buffer containing 50 mM Tris-HCl, pH 7.4. The radioactivity trapped was determined by liquid scintillation spectrometry. Data from at least five independent experiments were fitted by nonlinear regression analysis using GraphPad Prism. K_i_ values were calculated from the IC_50_ using the Cheng-Prusoff equation.

### Inhibition of cAMP Accumulation by LOR17 and U50,488

The activity of LOR and U50,488 was determined by measuring the inhibition of forskolin-stimulated cAMP accumulation in whole HEK-293/hKOPr cells, U87-MG cells and normal human astrocytes. Samples in a 75 cm^2^ flask at 95–100% confluence were split into 24 wells and incubated overnight. When the confluence became 85–95%, the medium was removed and the cells were washed three times with PBS; thereafter, cells were incubated in serum-free medium containing 0.5 mM 3-isobutyl-1-methylxanthine and exposed for 15 min to 10 μM forskolin, without and with each compound (0.001 nM to 100 μM) at 37°C. Cells were then lysed in 0.1 N HCl, scraped off, and centrifuged (2,000 g, 5 min). Supernatants were assayed for cAMP concentration using a cAMP EIA kit (Cayman Chemicals, Ann Arbor MI, USA) according to the manufacturer’s instructions.

### β-Arrestin 2 Recruitment

β-arrestin 2 recruitment at KOPr was investigated by using the DiscoveRxPathHunter^®^ eXpressβ-arrestin2 assay (Eurofins-DiscoveRx, CA, USA) according to manufacturer’s recommendations and previously published procedures (Zhou et al., 2013; Spetea et al., 2017). Briefly, U2OS cells provided with the kit and stably co-expressing the kappa receptor and the enzyme acceptor-tagged β-arrestin2 fusion protein were seeded in cell plating medium into 96-well plates (10,000 cells per well) and maintained for 48 h at 37°C. Cells were then exposed to vehicle, LOR17 (0–10 μM) or U50,488 (0–10 μM) and incubated for 3 h at 37°C; after that the detection mix was added and incubation was continued for further 60 min at room temperature in the dark. Chemiluminescence was measured *via* EnSpire^®^ Multimode Plate Reader (PerkinElmer Inc., Milan, Italy). All compounds were run in parallel assays in duplicate for comparison.

### BRET Assay

BRET-based assay with mYFP as the acceptor and RLuc as the donor was employed to investigate β-arrestin 2 recruitment at KOPr in HEK-293 cells exposed to vehicle, LOR17 (1–10 μM) or U50,488 (1–10 μM). BRET assay was carried out as previously reported (Gimenez et al., 2012; Bedini, 2015): briefly, HEK-293 cells were seeded into 6-well multiplates and transfected, using Lipofectamine^®^ 2,000 (Invitrogen) according to the manufacturer’s instructions (3 μl of Lipofectamine 2,000 per each μg of DNA), with increasing amounts of mYFP-β-arrestin 2 constructs (0–12 μg) along with 250 ng of KOPr-Luc encoding plasmid and empty pcDNA3 to equalize DNA amount. The day after transfection, cells were detached and plated into 96-well multiplates as previously described (Bedini, 2015). After 24 h cells were treated with vehicle, LOR17 (1–10 μM) or U50,488 (1–10 μM); then 5 μM coelenterazine-h was added and fluorescence and luminescence signals were detected *via* EnSpire^®^ Multimode Plate Reader (PerkinElmer Inc., Milan, Italy) at different time points between 5 and 60 min after ligands administration, according to the protocol previously reported (Bedini, 2015). The net BRET ratio was calculated as the long wavelength emission divided by the short wavelength emission and expressed as the relative change compared with unstimulated cells. The expression of mYFP-β-arrestin 2 was evaluated using fluorescence at 535 nm upon excitation at 485 nm. The mYFP-β-arrestin 2 fluorescence, which is directly proportional to the expression levels, was normalized by the basal luminescence from the respective KOPr-RLuc construct (F/L ratio) to account for variations in cell number and expression levels.

### Small Interfering RNA

β-arrestin-2 expression in U87-MG cells was knocked-down *via* a selective small interfering RNA (siRNA) as previously reported (Bedini et al., 2017). Briefly, siRNA for β-arrestin-2 or negative control siRNA (ctrl siRNA) were Silencer^®^ select pre-designed and validated siRNA obtained from Thermo Fisher Scientific (Walthman. Ma, USA). U87 cells were transfected with β-arrestin-2 siRNA or ctrl siRNA (50 nM) by using Lipofectamine^®^ RNAiMAX (Thermo Fisher Scientific) according to the manufacturer’s protocol and treatments were started 48 h later. The effectiveness of β-arrestin-2 silencing *via* siRNA was confirmed by western blot by using an anti-β-arrestin-2 antibody purchased from Abcam, according to the procedure that we previously employed (Bedini et al., 2017).

### Western Blot Analysis

HEK-293 cells stably expressing KOPr, U87-MG astrocytoma cells, or normal human astrocytes endogenously expressing KOPr were plated into 6-well plates until 60–70% confluence was reached. Then, the cells were serum-starved for 16–18 h and subsequently exposed to vehicle, LOR17 (1 μM; 0–120 min) or U50,488 (1 μM; 0–120 min). Alternatively, HEK-295/hKOPr and U87-MG cells were exposed to norBNI (10 μM; 30 min prior to LOR17) and then treated with 1 μM LOR17, or they were exposed to increasing concentration of LOR17 (1–100 μM; 30 min). Proteins were extracted as previously reported (Kuhar et al., 2015), quantified by means of BCA assay (Pierce) and SDS-page gel electrophoresis performed as previously described for ERK1/2 (Baiula et al., 2016) and p38MAPK (Schattauer et al., 2012).

Membranes were then incubated with anti-phospho-ERK 1/2 (1:1,000) (Cell Signaling Technology, Danvers, MA, USA), anti-total ERK1/2 antibodies (1:1,000) (Cell Signaling Technology), anti-phospho-p38MAPK antibodies (1:1,000) (Cell Signaling Technology, Danvers, MA, USA), or anti-actin antibodies (1:2,500) (Santa Cruz Biotechnology, Dallas, TX, USA) at 4°C in the presence of BSA (5 g/100 ml) in Tris buffered saline containing Tween-20 (0.1 g/100 ml). After washing, anti-rabbit horseradish peroxidase-conjugated secondary antibodies (Santa Cruz Biotechnology, Dallas, TX, USA) were added to membranes for 1.5 h at room temperature. The protocol of image acquisition and analysis has been previously described (Bedini et al., 2008) All the raw images of each representative western blot are included in the **Data Sheet 1**.

### Cell Proliferation Assay

A cell proliferation assay was carried out as previously reported (Bedini et al., 2017). Briefly, U87-MG cells or normal human astrocytes were plated on a 12-well plate and treated as described in the results section or maintained in cell culture medium containing 10% fetal bovine serum. Five hours before the end of the treatments, [methyl-^3^H] thymidine (Perkin Elmer) (50 nM final concentration) was added to serum-free cell culture medium, and the plate was incubated at 37°C. Thereafter, the medium was removed, and the cells were washed twice with PBS. Then, 200 μl of PBS was added to each well, and the cells were scraped off and centrifuged at 13,000 g for 3 min at 4°C. The supernatants were then discarded, pellets were re-suspended in 500 μl of cold trichloroacetic acid (10% w/v), incubated on ice for 20 min and centrifuged at 13,000 g for 3 min at 4°C. The supernatant was then discarded, and the pellet was suspended in 500 μl of cold methanol and centrifuged at 3 min for 13,000 g at 4°C. Thereafter, the pellet was suspended in 200 μl of 1 N NaOH and heated at 55°C for 10 min. Samples were then neutralized with 200 μl of 1 N HCl and 350 μl of the labelled DNA incubated in counting vials with 4 ml of Filter Count scintillation liquid (Perkin Elmer Italia). Vials were vortexed and incubated overnight at room temperature, and the radioactivity was determined by liquid scintillation spectrometry.

### Animals

CD-1 adult male mice (23–25 g) were used in the experiments. The animals were fed a standard laboratory diet and tap water ad libitum and housed in a room kept at 23 ± 1°C with a 12 h light/dark cycle (light on at 7 a.m.). All animal manipulations were carried out according to the Directive 2010/63/EU of the European parliament and of the European Union council (22 September 2010) on the protection of animals used for scientific purposes. The procedures regarding the warm-water tail-withdrawal test and writhing test employed in this study were approved by the Animal Care and Use Committee of the University of Bologna (Prot. n. 29-IX/9, 25th July 2012) and conformed to the International Association for the Study of Pain (IASP) guidelines on ethical standards for the investigation of experimental pain in animals. The ethical policy of the University of Florence complies with the Guide for the Care and Use of Laboratory Animals of the US National Institutes of Health (NIH Publication No. 85-23, revised 1996; University of Florence assurance number: A5278-01). Formal approval to conduct the experiments in the mouse model of oxaliplatin-induced neuropathy, rotarod test, hole-board test and forced swimming test was obtained from the Animal Subjects Review Board of the University of Florence. Experiments involving animals have been reported according to ARRIVE guidelines (McGrath and Lilley, 2015). All efforts were made to minimize animal suffering and to reduce the number of animals used.

### Warm-Water Tail-Withdrawal Test

The warm-water tail-withdrawal test was performed as previously described (Bedini et al., 2010). Briefly, a mouse’s tail was immersed in hot water (52 ± 0.5°C), and the latency to tail withdrawal was measured as an indicator of nociception. Prior to being treated, each mouse was tested, and the latency to tail withdrawal was recorded (control latency, CL). Animals not withdrawing their tails within 5 s were not used (6% of mice). Responding animals were then injected i.p. with either vehicle or one of the compounds (LOR17 or U50,488; 0–20 mg/kg). The KOR antagonist norBNI (10 mg/kg; i.p.) was injected 30 min or 24 h prior to administering LOR17.

Latency to withdrawal was measured at 5, 15, 30, 45, 60, and 90 min after drug administration and defined as the test latency (TL), with a cutoff time of 10 s. The antinociceptive response was expressed as the percentage of MPE = 100 × (TL − CL)/(10 − CL).

### Intraperitoneal Acetic Acid-Induced Writhing Test

Antinociception was evaluated in the different groups of mice placed individually in a large glass cylinder for observation by counting stretching or writhing responses during a 10-min period after an i.p. injection of AcOH acid (0.1 ml/10 g of a 0.6%, w/v solution in water). LOR17 or U50,488 (0–10 mg/kg) was i.p, administered 5 minutes before AcOH, and the control group was treated with an equal amount of vehicle (propylene glycol and saline, 1:1 ratio) before administering the AcOH solution.

### Oxaliplatin-Induced Cold Hypersensitivity Test and Pharmacological Treatments

Oxaliplatin neuropathy was induced in mice administered 2.4 mg/kg oxaliplatin intraperitoneally (i.p.) for 5 consecutive days each week for 2 weeks (Cavaletti et al., 2001; Brindisi et al., 2016). Oxaliplatin was dissolved in 5% glucose solution. Control animals received an equivalent volume of 5% glucose i.p. (vehicle). LOR17 was dissolved in 1:1 saline/propylene glycol solution, and U50,448 was dissolved in saline solution. For acute experiments, both compounds were administered subcutaneously (s.c.) on day 14 of the oxaliplatin treatment. Alternatively, LOR17 was administered s.c. daily starting on the first day of oxaliplatin injection. The cold plate test was performed on day 14 to assess the acute effects (from 15–75 min after the injection of LOR17 or U50,448) and on days 7 and 14 to evaluate the effects of repeated treatment (24 h after the last LOR17 administration). Eventual adverse effects induced by LOR17 were evaluated 30 min after a single administration of the compound (10 mg/kg, s.c.) in behavioral tests, namely, the hole-board, rotarod, and forced swimming tests.

### Cold Plate Test

The animals were placed in a stainless box (12 cm × 20 cm × 10 cm) with a cold plate as the floor. The temperature of the cold plate was kept constant at 4 ± 1°C. Pain-related behaviors (i.e., lifting and licking of the hind paw) were observed, and the time (s) until the first sign was recorded. The cutoff time for the latency to paw lifting or licking was set at 60 s (Carta et al., 2015).

### Rotarod Test

The integrity of the animals’ motor coordination was assessed, in a different group of mice, using a rotarod apparatus (Ugo Basile, Varese, Italy) at a rotating speed of 16 rpm. The treatment was performed before the test. The numbers of falls from the rod were counted for 30 s, before (0 min) and 15, 30, and 45 min after drug administration, and the test was performed according to the method described by Vaught et al. (1985).

### Hole-Board Test

The hole-board test consisted of a 40 cm^2^ plane with 16 flush-mounted cylindrical holes (3 cm diameter) distributed four by four in an equidistant, grid-like manner. Mice were placed on the center of the board one by one and allowed to move about freely for a period of 5 min. Two electric eyes, crossing the plane from midpoint to midpoint of the opposite sides, thus dividing the plane into four equal quadrants, automatically signaled the movement of the animal (counts in 5 min) on the surface of the plane (spontaneous motility). Miniature photoelectric cells in each of the 16 holes recorded (counts in 5 min) the exploration of the holes (exploratory activity) by the mice (Guerrini et al., 2006).

### Forced Swimming Test

The forced swimming test was carried out as described by Porsolt et al. (1977). Briefly, mice were placed individually into glass cylinders (height: 25 cm, diameter: 10 cm) containing 12 cm of water maintained at 22–23°C and left there for 6 min. A mouse was judged to be immobile when it floated in the water, in an upright position, and made only small movements to keep its head above water. The duration of mobility was recorded during the last 4 min of the 6-min test. An increase in the duration of mobility is indicative of an antidepressant-like effect.

### Blind Experiments

Researchers involved in the experiments, data collectors, outcome adjudicators, and data analysts that did participate to the study were blinded to reduce the risk of bias. Personnel who did not perform the experiments prepared the administered compounds.

### Materials

LOR17 was dissolved in a vehicle containing propylene glycol and saline in a 1:1 ratio.

U50,488 was obtained by Tocris and dissolved in ultrapure water for *in vitro* assays and in saline for *in vivo* experiments. Polyacrylamide gel, ammonium persulfate, sodium dodecyl sulfate, Tween 20, BSA, Triton-X-100, RNAlater, TRI Reagent, and Phosphatase Inhibitor Cocktail 3 were obtained from Sigma-Aldrich. Cell culture media, PBS, FBS, were from Thermo Fisher Scientific. Secondary antibodies for western blot analysis were from Santa Cruz Biotechnology (Santa Cruz, CA, USA), whereas antibodies directed toward MAPK were from Cell Signaling. Hybond-ECL nitrocellulose membrane was obtained from GE Healthcare (Chicago, USA). All the plastic disposables were from Sarstedt (Nümbrecht, Germany). All the other reagents were of analytical grade or the highest purity available and purchased from Sigma-Aldrich.

### Data and Statistical Analysis

All *in vitro* data represent the mean ± SD of at least five independent experiments, whereas for the *in vivo* warm-water tail-withdrawal and writhing tests, at least eight animals per group were analyzed. With regard to *in vitro* assays and *in vivo* acetic acid-induced writhing, statistical analysis was performed *via* GraphPad Prism 5.0 using one-way ANOVA and *post hoc* Newman-Keuls tests. With regard to warm-water tail-withdrawal test, statistical analysis was performed *via* GraphPad Prism 5.0 using two-way ANOVA. The data and statistical analysis comply with the recommendations on experimental design and analysis in pharmacology (Curtis et al., 2015). Regarding the oxaliplatin-induced neuropathy test, rotarod test, hole-board test, and forced swimming test, behavioral measurements were performed on 12 mice for each treatment carried out in 2 different experimental sets. A Bonferroni’s significant difference procedure was used as a *post hoc* comparison after ANOVA. IC_50_ and ED_50_ values were calculated from the respective concentration- or dose-dependent response curves *via* GraphPad Prism 5.0, by means of log(agonist)vs.response (three parametric) equation

## Results

### Peptide Synthesis, General Procedure

**Figure 1:**
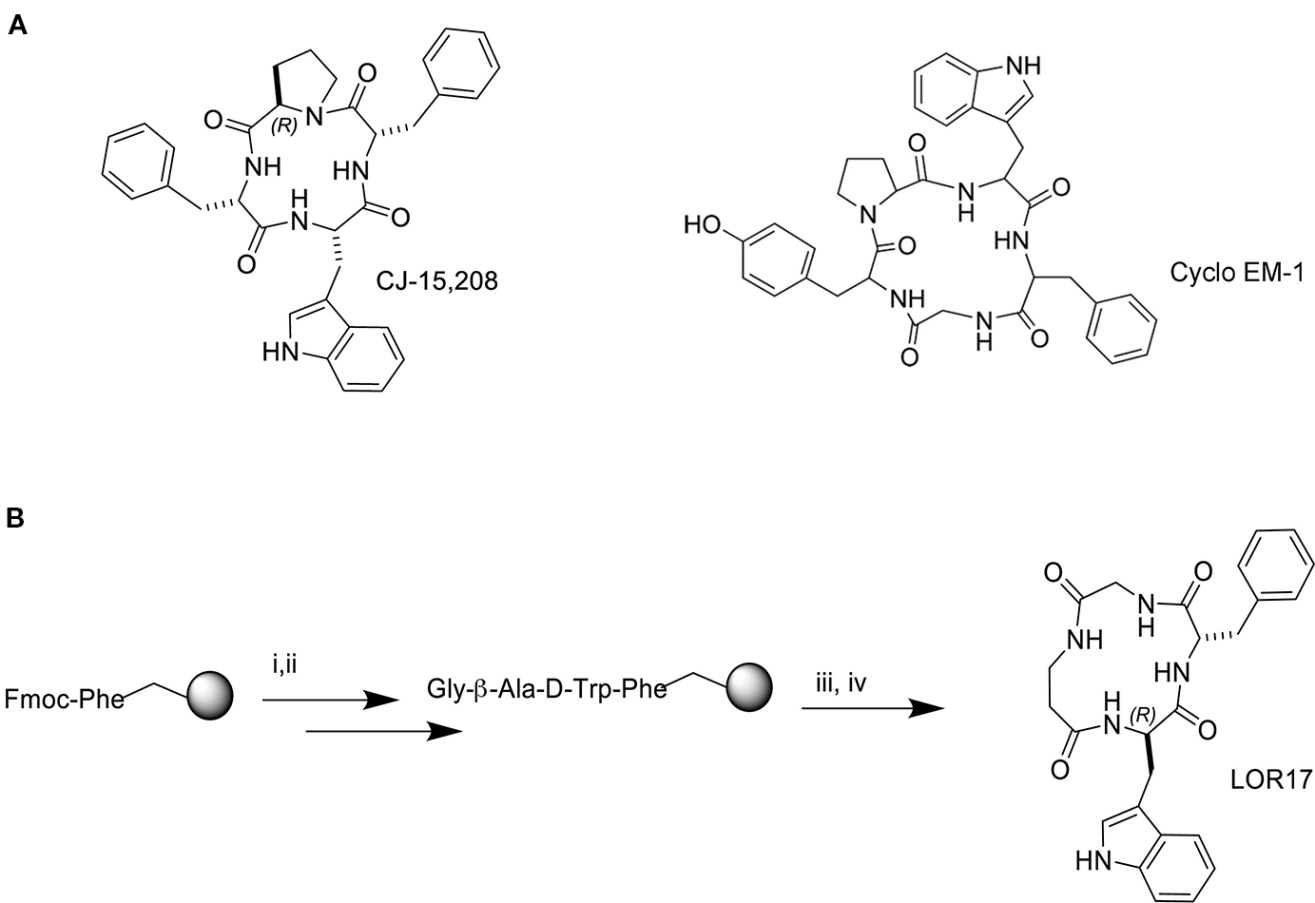
Structure of the natural peptide CJ-15,208 and the synthetic peptide Cyclo EM-1 **(A)** and synthesis of the peptide hybrid of the two sequences, LOR17 **(B)**; i. 20% Pip/DMF, ii. HOBt/TBTU/DIPEA, iii. TFA/TIPS/PhOH/water, iv. HATU/DIPEA/DMF.

The linear peptide precursor of LOR17 was synthesized on solid phase in the presence of TBTU/HOBt/DIPEA under conditions previously optimized (De Marco et al., 2017). Steps of Fmoc deprotection and coupling were repeated until the final linear tetrapeptide was obtained. Phe-Gly-β-Ala-D-Trp was recovered after cleavage with TFA and scavengers, and it was verified by analytical RP-HPLC (see General Procedures). The cyclization was performed under pseudo high dilution conditions using a temporized syringe in the presence of HATU/DIPEA/DMF, yielding c[Phe-Gly-β-Ala-D-Trp] (**Figure 1B**). The resulting crude residue was purified by semi-preparative RP HPLC (see *General Procedures*), and the cyclic peptide was characterized by reversed-phase HPLC, 1H NMR and 13C NMR. Purities were determined to be >95% by analytical RP-HPLC (see *General Procedures*) and elementary analysis.

### Binding Affinity of LOR17 to Different Opioid Receptors

LOR17 affinity to mu, delta and kappa receptors was evaluated by adopting a competition binding assay carried out in HEK-293/hMOPr, HEK-293/hDOPr, or HEK-293/hKOPr cells using [^3^H]DAMGO, [^3^H]-diprenorphine, or [^3^H]U69,593 as specific radioligands, respectively, as described in the Methods. The reference compounds DAMGO, DPDPE, and U50,488 showed K_i_ values in the nM range and high selectivity to the respective receptors (**Table 1**). LOR17 was a KOPr-selective ligand with a nanomolar affinity for the kappa receptor (**Table 1**).

**Table 1:** *In vitro* affinity of LOR17 and reference compounds to opioid receptors.

Compound	Sequence	K_i_ MOR (nM)	K_i_ DOR (nM)	K_i_ KOR (nM)
DAMGO	H-Tyr-D-Ala-Gly-NMePhe-Glyol	1.5 ± 0.1		
DPDPE	H-Tyr-c[D-Pen-Gly-Phe-D-Pen]-OH		3.30 ± 0.05	
U50,488	not peptide			2.90 ± 0.04
LOR17	c[Phe-Gly-(β-Ala)-D-Trp]	>10^5^	>10^5^	1.19 ± 0.28

Mean of 6 determinations ± SD.

### KOPr Expression and Inhibition of Forskolin-Induced cAMP Accumulation in HEK-293/hKOPr Cells, U87-MG Cells, and Primary Human Astrocytes

Prior to assessing LOR17 activity at the kappa receptor, KOPr density on the membranes of HEK-293/hKOPr cells, U87-MG cells and normal human astrocytes was quantified as an indirect measure of KOPr protein expression levels by means of saturation binding assays: kappa receptor density (B_Max_) was 1400 ± 220 fmol/mg in HEK-293/hKOPr cells, 412 ± 48 fmol/mg in U87-MG cells, and 326.3 ± 127.9 fmol/mg in normal human astrocytes (**Table 2**); KOPr expression, therefore, is significantly lower in the employed astrocyte cells as compared to HEK-293/KOPr cells (p < 0.001 vs HEK-293/hKOPR cells; Newman-Keuls test after ANOVA) (**Table 2**). The apparent affinity of [^3^H]-U69,593 to KOPr was similar in the three cell models, showing K_d_ values of 1.3 ± 0.3 nM in HEK-293/hKOPr cells, 2.2 ± 0.6 nM in U87-MG cells, and 2.0 ± 0.4 nM in normal human astrocytes (**Table 2**).

**Table 2:** Kappa opioid receptor (KOPr) density on cell membrane of different cell models.

Cell model	B_max_ (fmol/mg)	K_d_ (nM)
HEK-293/hKOPr	1400 ± 220	1.3 ± 0.3
U87-MG	412 ± 48^***^	2.2 ± 0.6
NHA	326.3 ± 127.85^***^	2.0 ± 0.4

Mean of 6 determinations ± SD; ***p < 0.001 vs HEK-293/hKOPr (Newman-Keuls test after ANOVA).

LOR17 activity as a KOPr agonist was investigated by measuring its ability to counteract forskolin-induced cAMP accumulation in HEK-293/KOPr cells, U87-MG human astrocytoma cells and normal human astrocytes. U50,488 was assayed under the same experimental conditions as a KOPr-selective reference agonist. As reported in **Table 3**, both U50,488 and LOR17 effectively inhibited forskolin-induced cAMP accumulation in all three cell models employed, displaying IC_50_ values in the nanomolar range and E_max_ values suggestive of full agonism (**Table 3**). Furthermore, LOR17 and U50,488 potency and efficacy in inhibiting forskolin-induced cAMP accumulation were not affected by the different KOPr expression levels observed in the three cell models employed.

**Table 3:** Inhibitory effects of LOR17 and U50,488 on forskolin-induced cAMP accumulation in different cell models.

COMPOUND	IC_50_ HEK-293/KOR (nM)	E_max_HEK-293/KOR (%)	IC_50_ U87-MG (nM)	E_max_U87-MG (%)	IC_50_ NHA (nM)	E_max_NHA(%)
U50,488	1.6 ± 0.5	90 ± 2	1.2 ± 0.2	88 ± 3	2.2 ± 0.4	87 ± 3
LOR17	2.8 ± 0.6	85 ± 5	3.1 ± 0.8	87 ± 4	3.0 ± 0.2	88 ± 6

Mean ± SD of 6 independent experiments performed in triplicate.

### LOR17 Biased KOPr Signaling Toward G Protein Activation Over β-Arrestin 2 Recruitment

**Figure 2:**
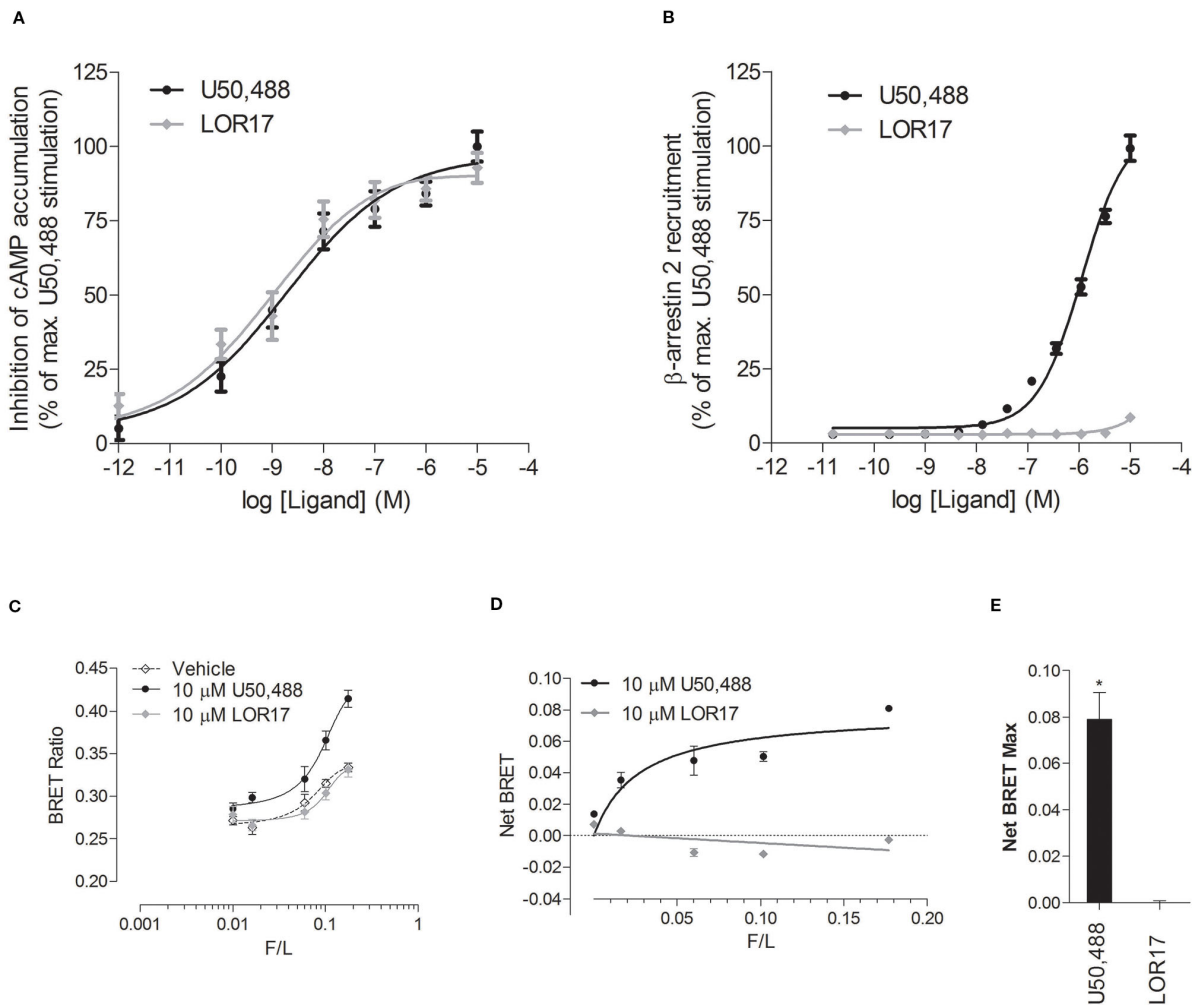
*In vitro* functional activity of LOR17 and U50,488. **(A)**. Inhibition of forskolin-induced cAMP accumulation assessed *via* cAMP ELISA in HEK-293/hKOPr cells. The data were normalized to the maximum stimulation caused by U50,488 (100%) (n = 5 independent experiments). **(B)**. Concentration–response curves of LOR17 and U50,488 for β-arrestin 2 recruitment to hKOPr expressed in U2OS–β-arrestin 2 cells using the PathHunter β-arrestin2 assay. Responses were normalized to the maximum effect of U50,488 (100%) (n = 3 independent experiments). All data are presented as the mean ± SD. **(C–E)**. BRET-based assays to assess the interaction of increasing amounts of arrestin 3-YFP with luciferase-tagged human KOPr. **(C)**. BRET ratio in the presence of U50,488, LOR17 or vehicle in HEK-293 cells co-expressing varying amounts of arrestin 3-YFP with RLuc-tagged KOPr; means ± S.D. of six repeats in a representative experiment (of five performed) are shown. **(D)**. Agonist-induced increase in BRET as a function of arrestin 3-YFP expression normalized by RLuc luminescence (F/L); Means ± S.D. of six repeats in a representative experiment (of five performed) are shown. **(E)**. Net BRET Max values calculated from U50,488 or LOR17 response curves plotted in panel D. *p < 0.05 vs LOR17 (t test).

To investigate LOR17 and U50,488 ability to recruit β-arrestin 2 at KOPr, ligands were tested within the PathHunter β-arrestin 2 recruitment assay with U2OS cells co-expressing hKOPr and the enzyme acceptor tagged β-arrestin 2 fusion protein. U50,488 was tested in parallel under the same conditions as a reference full KOPr agonist. We found that LOR17 induced a very week β-arrestin 2 recruitment at KOPr and only at concentrations higher than 1 μM (**Figure 2B**); on the other hand, both LOR17 and U50 determined a significant and similar inhibition of forskolin-induced cAMP accumulation in HEK-293/hKOPr cells (**Figure 2A**). Concentration-response curves obtained by using the two signaling assays above indicated were analyzed by using the operational model (van der Westhuizen et al., 2014; Stahl et al., 2015) in order to calculate the bias factor. Compared to U50,488, that was normalized to a value of 1, bias factor for LOR17 resulted equal to 853. The transduction ratio (log(τ/KA)) values for compounds and assays are listed in **Table 4**.

**Table 4:** Analysis of bias comparing G protein signaling and β-arrestin2 recruitment induced by LOR17 in comparison to U50,488 activity.

Compound	log(τ/K_A_)		Δlog(τ/K_A_)		ΔΔlog(τ/K_A_)	Bias Factor
	**G Protein^a^**	**β-arrestin 2^b^**	**G Protein^a^**	**β-arrestin 2^b^**		
U50,488	8,595	6,102	0	0	0	1
LOR17	8,856	3,432	0,261	-2,67	2,931	853

^a^Derived from the cAMP ELISA assay carried out in HEK-293 cells expressing the human KOP receptor (n = 5 independent experiments). ^b^Derived from the PathHunter β-arrestin2 recruitment assay with U2OS cells co-expressing the human KOP receptor and the enzyme acceptor tagged β-arrestin 2 fusion protein (n = 3 independent experiments). Transduction coefficients ((log(τ/KA), mean ± SEM), and bias factors (ΔΔlog(τ/KA)G protein - β-arrestin 2) were calculated using the operational model.

Furthermore, LOR17 and U50,488 ability to recruit β-arrestin 2 at KOPr was assessed also by means of a BRET assay carried out in HEK-293 cells as described in the *Methods*.

1 and 10 μM U50,488, but not 1 and 10 μM LOR17, significantly increased the BRET ratio signal as compared to vehicle treated cells, peaking the effect at 25 min of exposure (**Figure 2C**). Consistently, β-arrestin 2 demonstrated saturable agonist-induced binding to KOPr, measured as the difference between the BRET ratio in the presence and absence of agonist (net BRET) (**Figure 2D**), only when cells were exposed to U50,488 and not to LOR17; these results are reflected by the NetBRET_max_ values calculated from the Net Bret saturation curves (**Figure 2E**).

### Effects of U50,488 and LOR17 on ERK 1/2 and p38MAPK Phosphorylation

**Figure 3:**
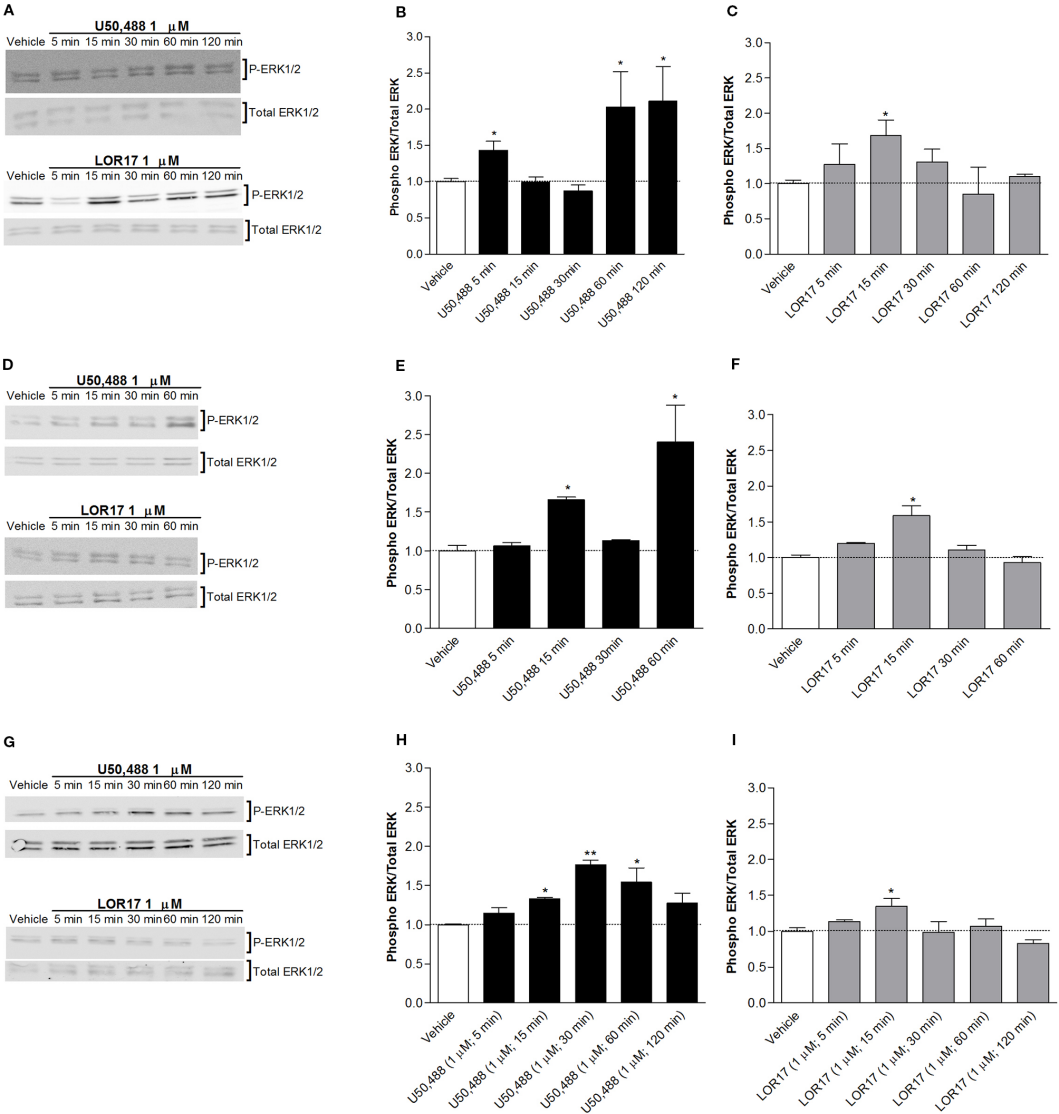
U50,488- and LOR17-mediated phospho-ERK1/2 (P-ERK1/2) increase in HEK-293/hKOPr cells, U87-MG astrocytoma cells and normal human astrocytes. **(A)**. Representative blots of HEK-293/hKOPr cells exposed to 1 µM U50,488, 1 µM LOR17 or vehicle. **(B**, **C)**. Quantification of P-ERK levels in HEK-293/hKOPr cells exposed to 1 µM U50,488, 1 µM LOR17 or vehicle; data are presented as the mean ± SD of 6 independent experiments. *p < 0.05 vs vehicle (Newman-Keuls test after ANOVA). **(D)**. Representative blots of U87-MG human astrocytoma cells exposed to 1 µM U50,488, 1 µM LOR17 or vehicle. **(E**, **F)**. Quantification of P-ERK levels in U87-MG human astrocytoma cells exposed to 1 µM U50,488, 1 µM LOR17 or vehicle; data are presented as the mean ± SD of 6 independent experiments. *p < 0.05 vs vehicle (Newman-Keuls test after ANOVA). **(G)**. Representative blots of normal human astrocytes exposed to 1 µM U50,488, 1 µM LOR17 or vehicle. **(H, I)**. Quantification of P-ERK levels in normal human astrocytes exposed to 1 µM U50,488, 1 µM LOR17 or vehicle; data are presented as the mean ± SD of 6 independent experiments. *p < 0.05 vs vehicle; **p < 0.01 vs vehicle (Newman-Keuls test after ANOVA).

U50,488- and LOR17-mediated ERK1/2 and p38MAPK phosphorylation was investigated in HEK-293/hKOPr cells, U87-MG cells and human astrocytes by western blot analysis. U50,488 (1 µM) significantly increased ERK1/2 phosphorylation levels in all three cell models mentioned above, with peaks of ERK1/2 activation at 5, 60, and 120 min in HEK-293/hKOR cells (**Figures 3A, B**) (p < 0.05 vs Vehicle; Newman-Keuls test after ANOVA), at 15 and 60 min in U87-MG cells (**Figures 3D, E**) (p < 0.05 vs Vehicle; Newman-Keuls test after ANOVA) and at 15, 30, and 60 min in human astrocytes (**Figures 3G, H**).

LOR17 (1 µM) produced a significant increase in ERK1/2 phosphorylation only after 15 min of exposure; this effect was similar in all three cell models employed in this study (**Figures 3A, C, D, F, G, I**) (p < 0.05 vs Vehicle; Newman-Keuls test after ANOVA).

Moreover, LOR17 (1μM)-mediated increase in ERK1/2 phosphorylation in HEK-293/hKOPr and U87-MG cells was fully reverted by pre-administering the KOPr antagonist norBNI (10 μM; 30 min prior to LOR17) (**Figures 5A, B**).

**Figure 4:**
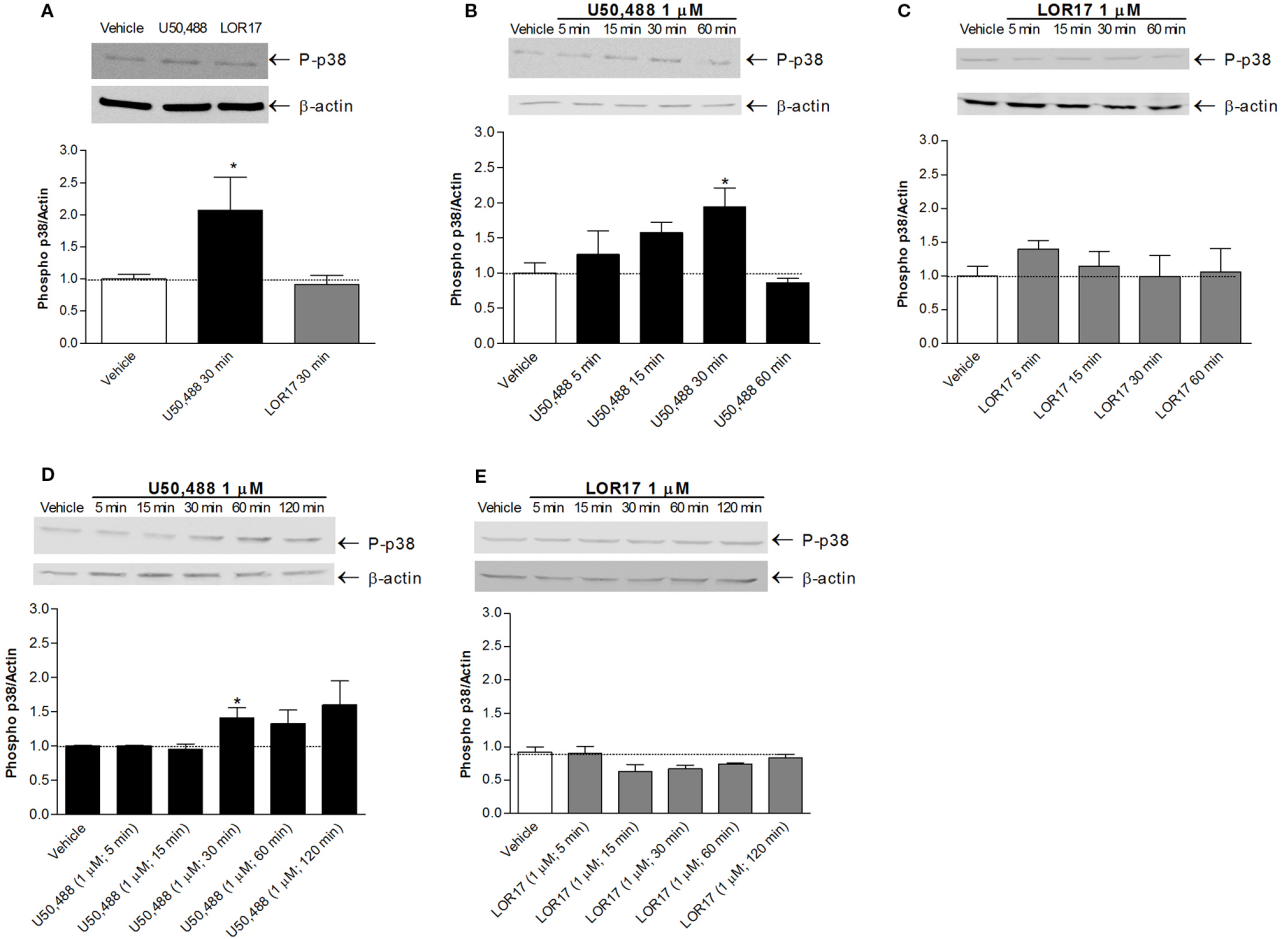
U50,488 and LOR17-mediated phospho-p38MAPK (P-p38) increase in HEK-293/hKOPr cells, U87-MG astrocytoma cells and normal human astrocytes. **(A)**. Representative blots and quantification of P-p38 levels in HEK-293/hKOPr cells exposed to U50,488 (1 µM; 30 min), LOR17 (1 µM; 30 min) or vehicle; data are presented as the mean ± SD of 6 independent experiments. *p < 0.05 vs vehicle (Newman-Keuls test after ANOVA). **(B**, **C)**. Representative blots and quantification of P-p38 levels in U87-MG human astrocytoma cells exposed to 1 µM U50,488, 1 µM LOR17 or vehicle; data are presented as the mean ± SD of 6 independent experiments. *p < 0.05 vs vehicle (Newman-Keuls test after ANOVA). **(D**, **E)**. Representative blots and quantification of P-p38 levels in normal human astrocytes exposed to 1 µM U50,488, 1 µM LOR17 or vehicle; data are presented as the mean ± SD of 6 independent experiments. *p < 0.05 vs vehicle (Newman-Keuls test after ANOVA).

**Figure 5:**
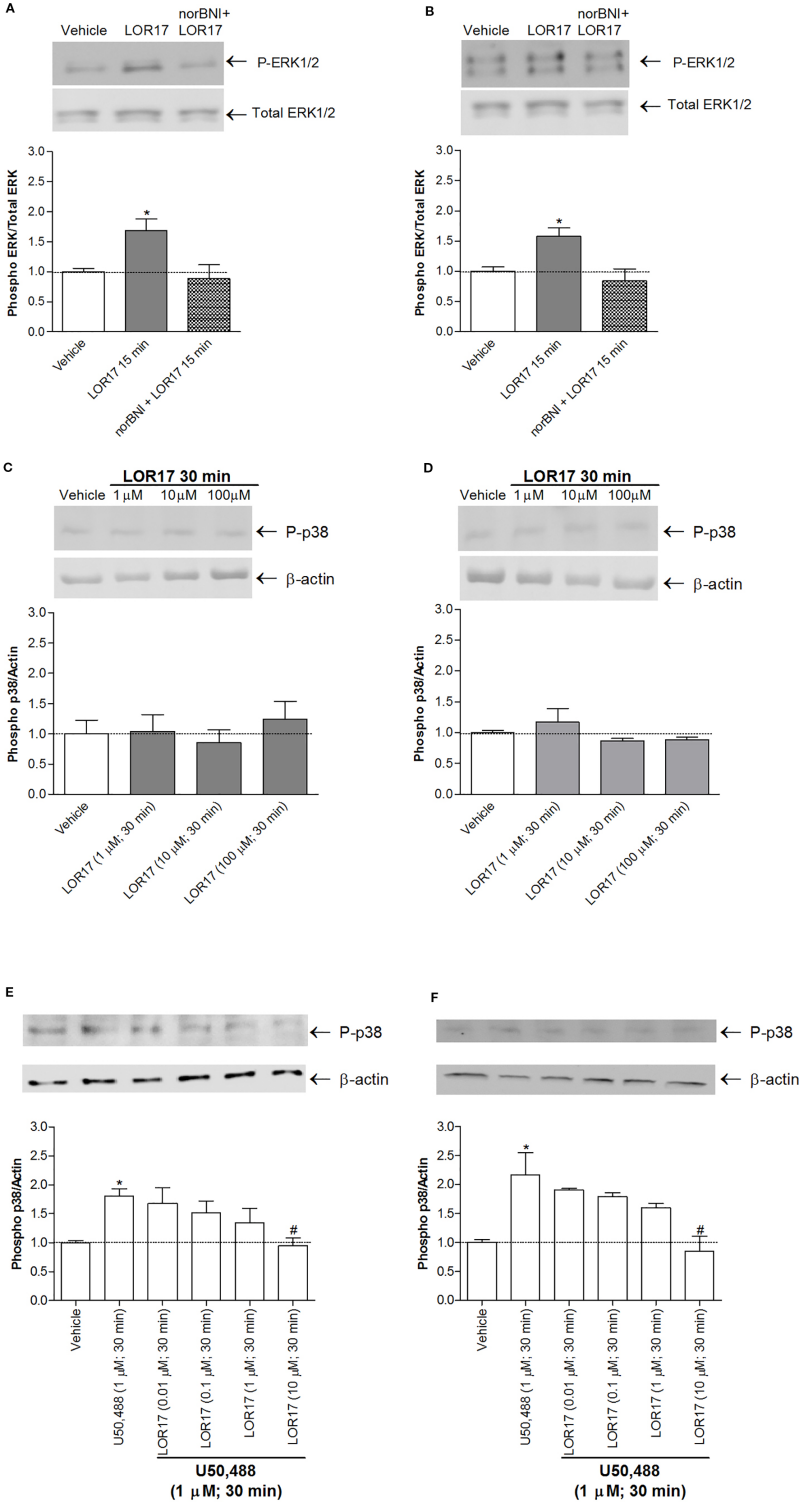
LOR17 increased P-ERK levels in a KOPr-dependent way and did not increase P-p38 levels in HEK-293/hKOPr and U87-MG cells, even when administered at concentrations up to 100 μM. **(A)**. Representative blots and quantification of P-ERK levels in HEK-293/hKOPr cells exposed to norBNI (10 µM; 30 min prior to LOR17) and LOR17 (1 µM; 15 min), LOR17 (1 μM; 15 min) alone or vehicle; data are presented as the mean ± SD of 6 independent experiments.*p < 0.05 vs vehicle (Newman-Keuls test after ANOVA). **(B)**. Representative blots and quantification of P-ERK levels in U87-MG cells exposed to norBNI (10 µM; 30 min prior to LOR17) and LOR17 (1 µM; 15 min), LOR17 (1 μM; 15 min) alone or vehicle; data are presented as the mean ± SD of 6 independent experiments. *p < 0.05 vs vehicle (Newman-Keuls test after ANOVA). **(C)**. Representative blots and quantification of P-p38 levels in HEK-293/hKOPr cells exposed to LOR17 (1-100 µM; 30 min) or vehicle; data are presented as the mean ± SD of 6 independent experiments. **(D)**. Representative blots and quantification of P-p38 levels in U87-MG cells exposed to LOR17 (1-100 µM; 30 min) or vehicle; data are presented as the mean ± SD of 6 independent experiments. **(E)**. Representative blots and quantification of P-p38 levels in HEK-293/hKOPr cells exposed to vehicle, U50,488 (1 µM; 30 min) alone or co-administered with LOR17 (0.01-10 µM; 30 min); data are presented as the mean ± SD of 6 independent experiments. **(F)**. Representative blots and quantification of P-p38 levels in U87-MG cells exposed to vehicle, U50,488 (1 µM; 30 min) alone or co-administered with LOR17 (0.01-10 µM; 30 min); data are presented as the mean ± SD of 6 independent experiments. *p < 0.05 vs vehicle; ^#^p < 0.05 vs U50,488 (Newman-Keuls test after ANOVA).

Regarding p38MAPK phosphorylation, it was significantly enhanced in HEK-293/hKOPr cells, U87-MG cells and human astrocytes after 30 min of exposure to 1 µM U50,488 (p < 0.05 vs Vehicle; Newman-Keuls test after ANOVA) but not to 1 µM LOR17. Furthermore, the latter does not influence p38MAPK phosphorylation either at any of the time points chosen (**Figure 4**) or at concentrations up to 100 μM (**Figures 5C, D**). Moreover, LOR17 counteracted U50,488-induced p38MAPK phosphorylation in a concentration-dependent fashion (**Figures 5E, F**).

### U50,488, but Not LOR17, Promotes U87-MG and Human Astrocyte Cell Proliferation in a KOPr-Dependent, β-Arrestin 2- and p38MAPK-Mediated Fashion

**Figure 6:**
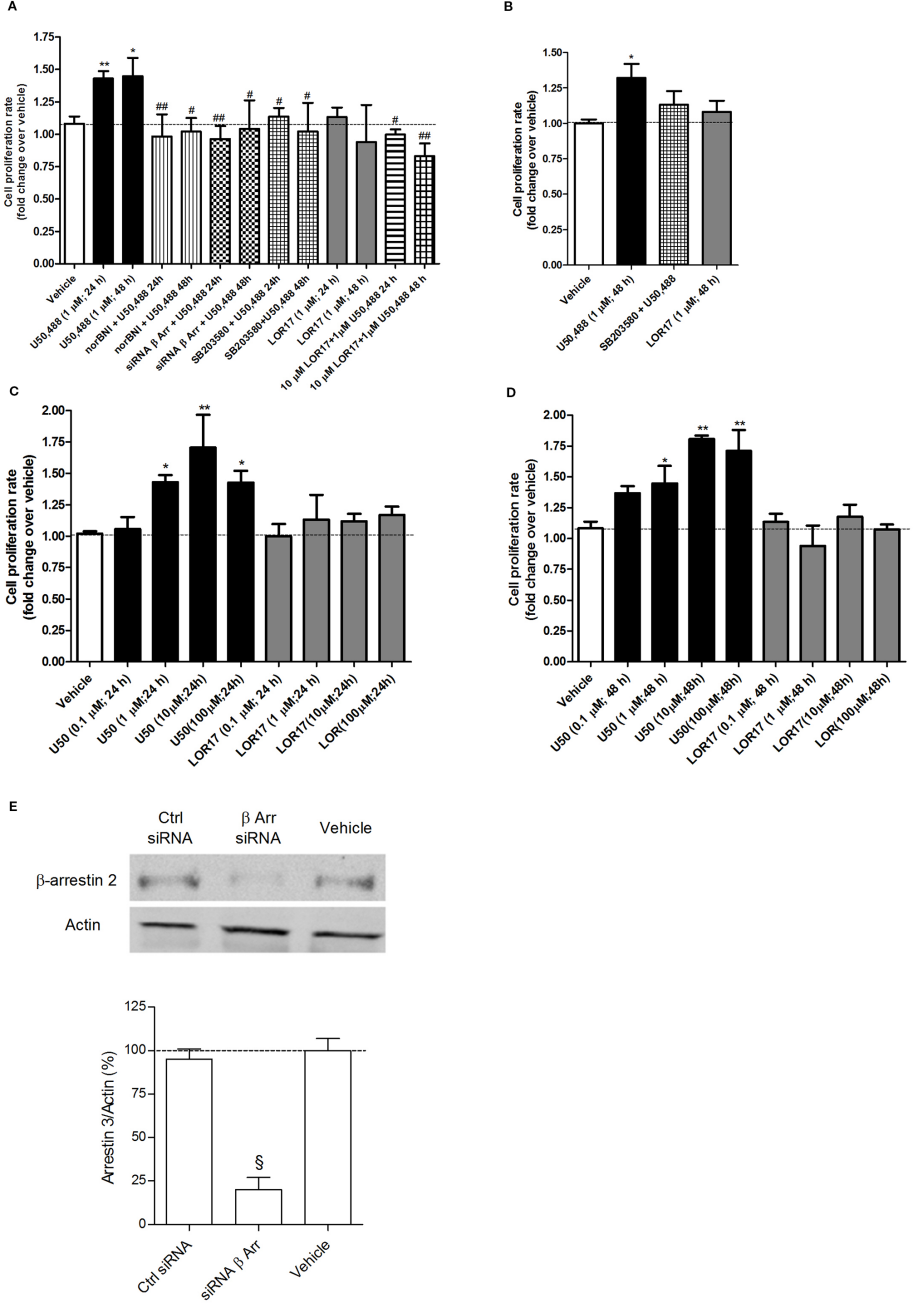
U50,488- and LOR17-mediated effects on cell proliferation of U87-MG human astrocytoma cells and normal human astrocytes. **(A)**. U50,488 (1 µM) produced a KOPr-dependent, β-arrestin 2- and p38MAPK-mediated increase in the U87-MG cell proliferation rate, whereas LOR17 did not; LOR17 counteracted U50,488-mediated induction of U87-MG cell proliferation. Data are presented as the mean ± SD of 8 independent experiments. *p< 0.05 vs vehicle, LOR17 (1 µM; 24 h) and LOR17 (1 µM; 48 h); **p< 0.01 vs vehicle, LOR17 (1 µM; 24 h) and LOR17 (1 µM; 48 h); ^#^p< 0.05 vs U50,488 (1 µM; 24 h) and U50,488 (1 µM; 48 h); ^##^p< 0.01 vs U50,488 (1 µM; 24 h) and U50,488 (1 µM; 48 h) (Newman-Keuls test after ANOVA). **(B)**. U50,488 (1 µM) resulted in a p38MAPK-mediated increase in the normal human astrocyte cell proliferation rate, whereas LOR17 did not. Data are presented as the mean ± SD of 8 independent experiments. *p < 0.05 vs vehicle, LOR17 (1 µM; 48 h) and SB203580 + U50,488 (Newman-Keuls test after ANOVA). **(C**, **D)**. U50,488 (0.1-100 µM) produced a concentration-dependent increase in the U87-MG cell proliferation rate at both 24 and 48 h of exposure, whereas LOR17 (0.1-100 µM) did not. Data are presented as the mean ± SD of 8 independent experiments. *p< 0.05 vs vehicle; **p < 0.01 vs vehicle (Newman-Keuls test after ANOVA). **(E)**. Knock-down of beta-arrestin 2 expression in U87-MG cells by means of selective siRNA. Data are presented as the mean ± SD of 6 independent experiments. ^§^p < 0.001 vs Vehicle and Ctr siRNA (Newman-Keuls test after ANOVA).

It has been clearly demonstrated in animal models of neuropathic pain (i.e., partial sciatic nerve ligation, partial infraorbital nerve ligation, streptozotocin-induced pain) that KOPr-dependent p38MAPK activation by endogenous dynorphin may promote astrocyte activation and proliferation, thus contributing to allodynia and hyperalgesia. Moving from these considerations, the influence of U50,488 and LOR17 on astrocyte cell proliferation was investigated *via* a [^3^H]-thymidine incorporation assay in U87-MG cells and human astrocytes. Concentration- and time-dependent responses were evaluated: U50,488 (0–100 µM) induced a significant and concentration-dependent increase in the U87-MG cell proliferation rate at both 24 h and 48 h (p < 0.05 vs Vehicle; Newman-Keuls test after ANOVA), whereas LOR17 (0–100 µM) did not (**Figures 6C, D**).

The U50,488 (1 µM)-mediated increase of U87-MG cell proliferation following 24 h and 48 h of exposure was prevented by the KOPr-selective antagonist norBNI (10 µM; 15 min prior to U50,488) as well as by siRNA selective for β-arrestin 2 (50 nM; 48 h) or by the p38MAPK inhibitor SB203580 (2 µM; 30 min prior to U50,488) (**Figure 6A**) (p < 0.05 vs U50,488; Newman-Keuls test after ANOVA). In contrast, LOR17 (1 µM) did not alter the U87-MG cell proliferation rate at either 24 h or 48 h of exposure (**Figure 6A**), not even when administered at concentration up to 100 μM (**Figures 6C, D**). Moreover, when 10 μM LOR17 was co-administered together with 1 μM U50,488, the former prevented the increase in U87-MG cell proliferation induced by the latter (**Figure 6A**).

Similarly, 1 µM U50,488, but not 1 µM LOR17, significantly enhanced normal human astrocyte cell proliferation (p < 0.05 vs Vehicle; Newman-Keuls test after ANOVA), and this effect was abolished by the p38MAPK inhibitor SB203580 (2 µM; 30 min prior to U50,488) (**Figure 6B**) (p < 0.05 vs U50,488; Newman-Keuls test after ANOVA). The β-arrestin 2 selective siRNA significantly knocked-down β-arrestin 2 expression in U87-MG cells, as confirmed by western blot (**Figure 6E**) (p < 0.05 vs Vehicle and Ctrl siRNA; Newman-Keuls test after ANOVA).

### U50,488 and LOR17 Promote Antinociceptive Effects in Different Models of Nociceptive Pain

U50,488- and LOR17-mediated antinociception was investigated in adult male mice *via* the warm-water tail-withdrawal test and by the writhing test, as described in the *Methods*.

**Figure 7:**
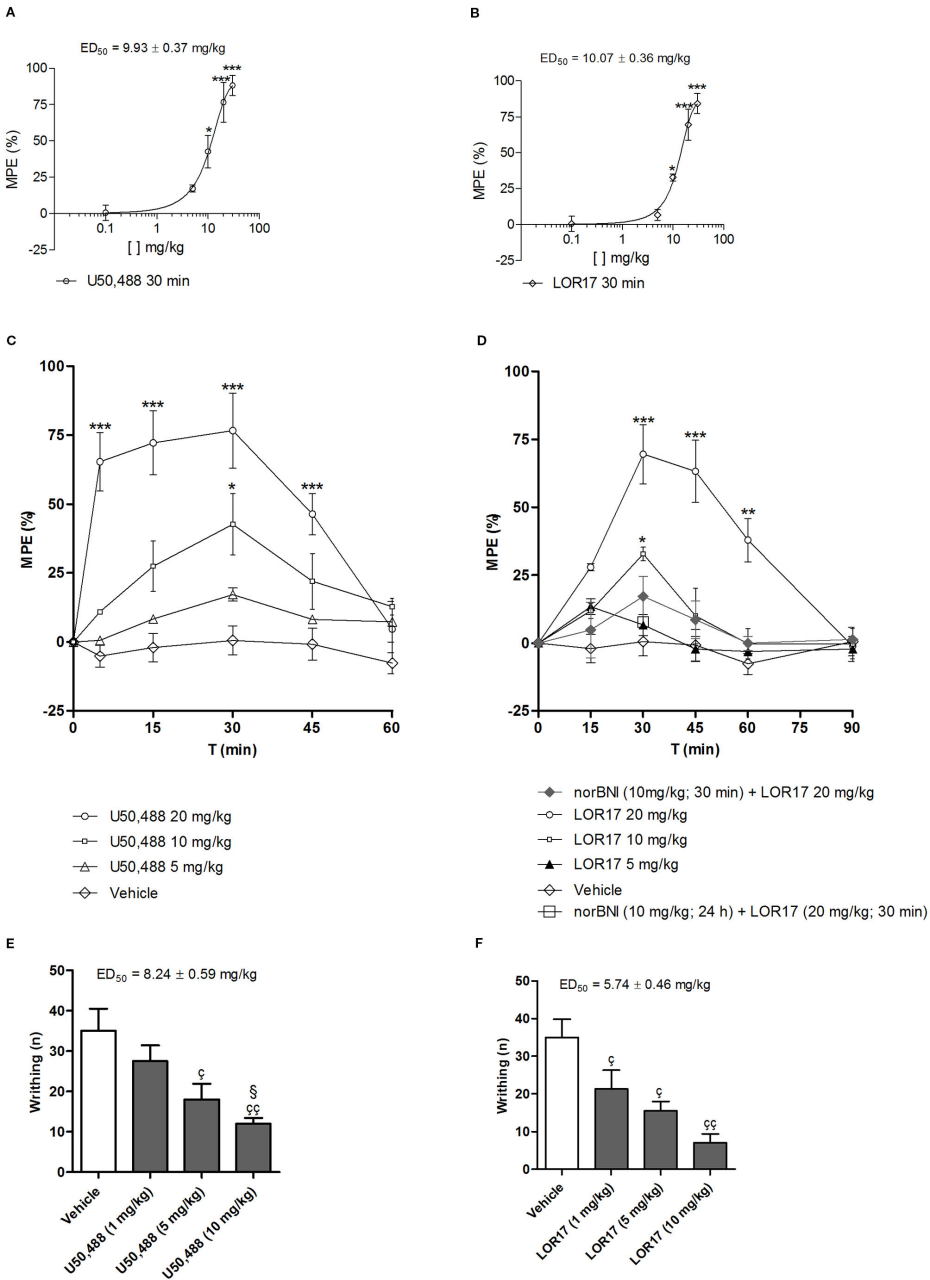
Antinociceptive effects mediated by U50,488 and LOR17 in mice; vehicle (saline for U50,488 and 1:1 mixture of propylene glycol and saline for LOR17), U50,488 or LOR17 were administered i.p. **(A)**. Dose-response curve of the antinociceptive effect induced in the warm-water tail-withdrawal test by i.p. administered U50,488. Data are presented as the mean ± SD of 10 mice. *p < 0.05 vs vehicle and U50,488 5 mg/kg; ***p < 0.001 vs vehicle and U50,488 5 mg/kg (Newman-Keuls test after ANOVA). **(B)**. Dose-response curve of the antinociceptive effect induced in the warm-water tail-withdrawal test by i.p. administered LOR17. Data are presented as the mean ± SD of 10 mice. *p < 0.05 vs vehicle and LOR17 5 mg/kg; ***p < 0.001 vs vehicle and LOR17 5 mg/kg (Newman-Keuls test after ANOVA). **(C)**. U50,488 produced a time- and dose-dependent antinociception in the warm-water tail-withdrawal test. Data are presented as the mean ± SD of 10 mice. *p < 0.05 vs vehicle and U50,488 5 mg/kg; ***p < 0.001 vs vehicle and U50,488 5 mg/kg (Newman-Keuls test after ANOVA). **(D)**. LOR17 determined a time- and dose-dependent, KOPr-mediated antinociception in the warm-water tail-withdrawal test. Data are presented as the mean ± SD of 10 mice. *p < 0.05 vs vehicle and LOR17 5 mg/kg; **p < 0.01 vs vehicle; ***p < 0.001 vs vehicle, LOR17 5 mg/kg, norBNI 10 mg/kg + LOR17 20 mg/kg and norBNI (10 mg/kg; 24 h) + LOR17 (20 mg/kg; 30 min**p < 0.01 vs vehicle) (Newman-Keuls test after ANOVA). **(E)**. U50,488 dose-dependently reduced the number of abdominal writhes induced in mice after i.p. injection of 0.6% acetic acid. Data are presented as the mean ± SD of 10 mice. ^ç^p < 0.05 vs vehicle; ^çç^p < 0.01 vs vehicle; ^§^p< 0.05 vs 1 mg/kg (Newman-Keuls test after ANOVA). **(F)**. LOR17 dose-dependently reduced the number of abdominal writhes induced in mice after an i.p. injection of 0.6% acetic acid. Data are presented as the mean ± SD of 10 mice. ^ç^p < 0.05 vs vehicle; ^çç^p < 0.01 vs vehicle (Newman-Keuls test after ANOVA).

U50,488 and LOR17 display similar dose-response curves at 30 min after i.p. injection, yielding to comparable ED_50_ values in the warm-water tail-withdrawal test (**Figures 7A, B**). In the warm-water tail-withdrawal test, ED_50_ was equal to 10.07 ± 0.36 mg/kg (95% C.I. 9.82–10.32 mg/kg) for LOR17 and to 9.93 ± 0.37 mg/kg (95% C.I. 9.66–10.19 mg/kg) for U50,488; in the writhing test, ED_50_ was equal to 5.74 ± 0.46 mg/kg (95% C.I. 5.41–6.07 mg/kg) for LOR17 and to 8.24 ± 0.59 mg/kg (95% C.I. 7.82–8.66 mg/kg) for U50,488.

U50,488 (0–20 mg/kg, 0–60 min; i.p.) produced a significant, dose- and time-dependent antinociception in the tail-withdrawal test (**Figure 7C**). LOR17 (0–20 mg/kg, 0–90 min; i.p.) promoted an antinociceptive response in a dose- and time-dependent fashion, displaying its effects starting at 30 min after injection and lasting up to 60 min after administration (at least with regard to the highest dose employed) (**Figure 7D**). LOR17-mediated antinociception was prevented by i.p. administration of the KOPr-selective antagonist norBNI (10 mg/kg) (**Figure 7D**).

Abdominal contractions induced upon i.p. injection of 0.6% acetic acid were adopted as a measure of visceral pain; within this experimental setting, pre-emptive i.p. administration of both U50,488 and LOR17 (0–10 mg/kg) significantly reduced the number of writhes (**Figures 7E, F**) (p < 0.05 vs Vehicle; Newman-Keuls test after ANOVA).

### LOR17, but Not U50,488, Significantly Reduces Thermal Hypersensitivity in a Mouse Model of Oxaliplatin-Induced Neuropathic Pain After Both Single and Repeated s.c. Administration

**Figure 8:**
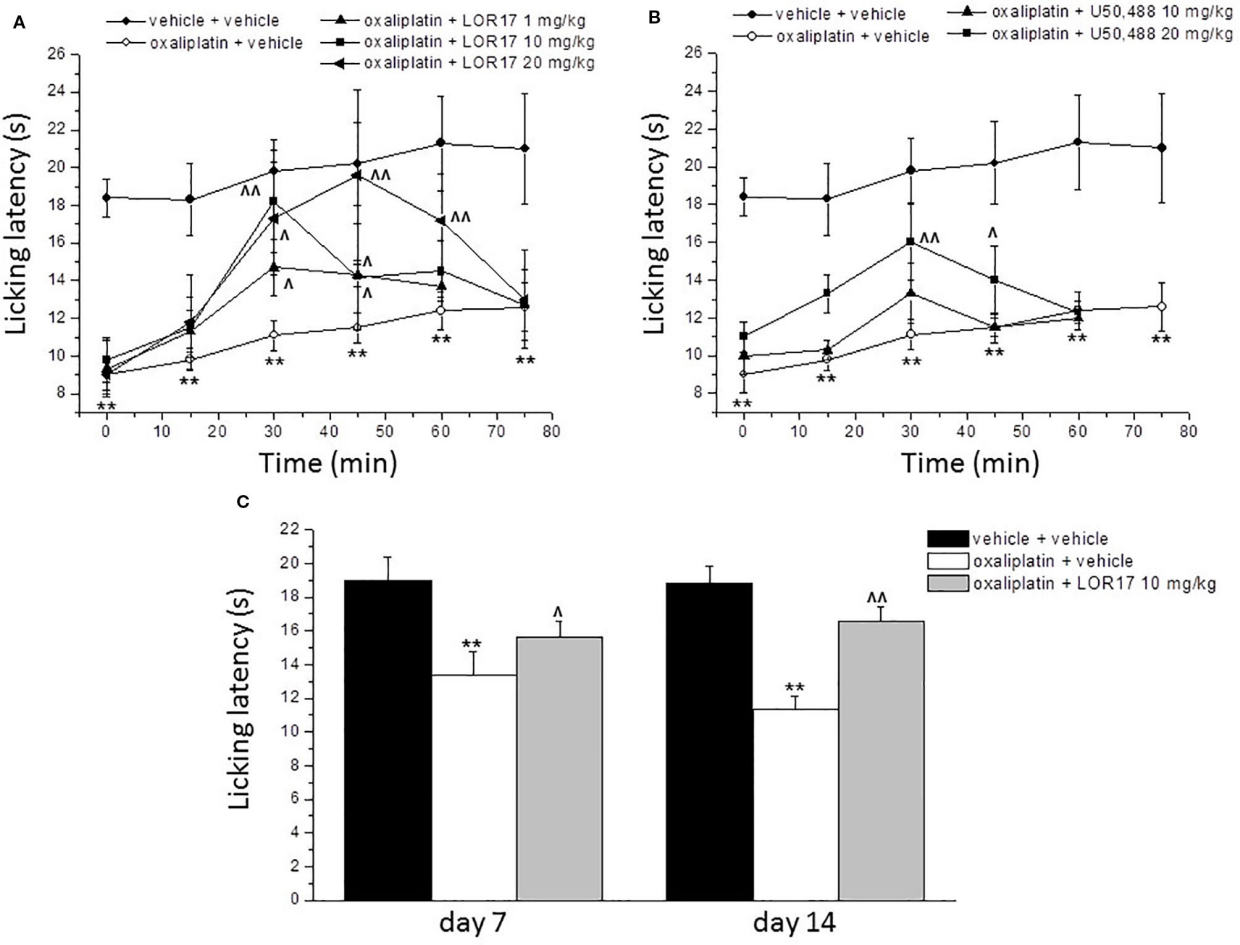
Effect of LOR17 on oxaliplatin-induced neuropathic pain. **(A)**. Acute treatment: on day 14 of oxaliplatin administration (2.4 mg/kg, i.p., administered daily), LOR17 (1 – 20 mg/kg) was administered s.c. The response to a thermal non-noxious stimulus was evaluated over time by the cold plate test measuring the latency to pain-related behaviors (lifting or licking of the paw). **(B)**. LOR17 effects were compared with those induced by the acute administration of U50,448 (10 and 20 mg/kg, s.c.). **(C)**. Repeated treatment: LOR17 (10 mg/kg) was administered s.c. daily starting the first day of oxaliplatin treatment. Cold hypersensitivity (cold plate test) was performed on days 7 and 14. Each value represents the mean ± S.E.M. of 12 mice performed in 2 different experimental set. **P < 0.01 versus vehicle + vehicle treated animals; ^^^ *P* < 0.05 versus oxaliplatin + vehicle; ^^^^ *P* < 0.01 versus oxaliplatin + vehicle (Bonferroni test after ANOVA).

A cumulative dose of oxaliplatin induces the development of a painful neuropathy as evaluated by the measurement of the response to a thermal stimulus. On day 14 of oxaliplatin treatment, mice injected with oxaliplatin showed a decrease in the pain threshold evaluated by the cold plate test (**Figure 8**). A single administration of s.c. LOR17 increased the licking latency in a dose-related manner, taking effect from 1 mg/kg (**Figure 8A**). At doses of 10 and 20 mg/kg, LOR17 fully reversed the oxaliplatin-dependent hypersensitivity, peaking between 30 and 45 min after s.c. administration. Conversely, U50,488 could significantly reduce, at least in part, oxaliplatin-induced thermal hypersensitivity when administered at 20 mg/kg (**Figure 8B**). However, the maximal effect elicited by U50,488, when administered at 10 or 20 mg/kg, was significantly lower as compared to the maximal effect induced by similar doses of LOR17 (**Table 5**). Furthermore, this newly synthesized KOPr agonist had an ED_50_ value of 6.63 ± 0.23 mg/kg (95% C.I. 6,49–6,77 mg/kg); on the other hand, it was not possible to calculate the corresponding ED_50_ value for U50,488 administered up to 20 mg/kg.

**Table 5:** Maximal effects elicited by LOR17 and U50,488 in counteracting oxaliplatin induced thermal hypersensitivity, as compared to vehicle.

Treatment	Licking latency (s)
oxaliplatin/vehicle	11.4 ± 1.2
oxaliplatin/U50,488 (10 mg/kg; 30 min)	12.6 ± 1.7
oxaliplatin/LOR17 (10 mg/kg; 30 min)	18.2 ± 1.4^***^
oxaliplatin/U50,488 (20 mg/kg; 30 min)	15.8 ± 1.6
oxaliplatin/LOR17 (20 mg/kg; 45 min)	19.6 ± 1.7^***^

On day 14 of oxaliplatin administration (2.4 mg/kg, i.p., administered daily), LOR17 or U50,488 (10 and 20 mg/kg) were administered s.c. The response to a thermal non-noxious stimulus was evaluated over time by the cold plate test measuring the latency to pain-related behaviors (lifting or licking of the paw). Maximal effects elicited in the dose-response curve (see **Figures 8A, B**) are reported as compared to vehicle. Data are presented as the mean ± SD of 10 mice. ***p < 0.001 vs oxaliplatin/vehicle, oxaliplatin U50,488 (10 mg/kg; 30 min), oxaliplatin/U50,488 (20 mg/kg; 30 min); n = 12 (Newman-Keuls test after ANOVA).

Aimed at evaluating the pain-relieving profile of LOR17 following repeated treatments, this compound (10 mg/kg) was injected s.c. daily starting on the first day of oxaliplatin administration. This dose was selected as it was the lowest dose giving rise to the maximal effect, as assessed after acute LOR17 administration. The cold plate test assessed on days 7 and 14, 24 h after the administration of LOR17, revealed that LOR17 maintains its efficacy (**Figure 8C**); thus, dispelling any development of tolerance.

### LOR17 Does Not Alter Motor Coordination, Locomotor, and Exploratory Activities and Does Not Induce Pro-Depressant-Like Behavior

The relieving effect of LOR17 in oxaliplatin-induced thermal hypersensitivity is free from behavioral side effects. Motor coordination (**Table 6**, rotarod test) as well as locomotor and exploratory activities (**Table 6**; hole-board test) were unaltered after LOR17 administration (10 mg/kg, s.c.). Finally, LOR17 (10 mg/kg, s.c.) did not induce pro-depressant-like behavior as evaluated by the forced swimming test (**Table 6**). Conversely, 10 mg/kg U50,488 significantly altered rotarod performance and reduced mobility time in the Prosolt test (**Table 6**). The dose of 10 mg/kg was selected as it was the lowest dose giving rise to the LOR17-mediated maximal effect in the oxaliplatin-induced neuropathic mice, as assessed after acute LOR17 administration; in fact, U50,488 administered at the same dose was not effective in relieving oxaliplatin-induced thermal hypersensitivity.

**Table 6:** Effect of LOR17 on motor coordination (rotarod test^a^), locomotor, and exploratory activitivities (hole-board test^b^) and pro-depressant like behaviour (forced swimming test^c^).

Treatment	Dose *mg kg^-1^ s.c.*	Number of falls^a^				Hole^b^		Board^b^		Mobility time (s)^c^
		0 min	15 min	30 min	45 min	0 min	30 min	0 min	30 min	
vehicle		3.0 ± 0.6	2.3 ± 0.3	1.8 ± 0.6	1.3 ± 0.3	42.4 ± 4.6	48.2 ± 4.1	81.6 ± 13.1	106.2 ± 8.0	80.5 ± 9.6
LOR17	10	2.8 ± 0.7	1.6 ± 0.4	1.2 ± 0.2	0.6 ± 0.2	37.2 ± 3.9	39.0 ± 4.6	89.2 ± 7.3	97.2 ± 16.9	91.6 ± 12.3
U50,488	10	2.9 ± 0.4	3.2 ± 0.5^§^	2.6 ± 0.3^§§^	2.1 ± 0.4^§§^	/	/	/	/	21.0 ± 7.7 ^***^

a Rotarod test: LOR17 was dissolved in 1:1 saline solution/propylene glycol mixture and subcutaneously administered. The integrity of motor coordination of the mouse was assessed on the basis of the number of falls from the rod in 30 s The performance time was measured before (0 min) and 15, 30, and 45 min after the beginning of the test. § p < 0.05 vs vehicle and LOR17; ^§§^p < 0.01 vs vehicle and LOR17; n = 12 (Newman-Keuls test after ANOVA).

b Hole-board test: LOR17 was dissolved in 1:1 saline solution/propylene glycol mixture and subcutaneously administered 25 min before the beginning of the test. Two electric eyes, crossing the plane from midpoint to midpoint of opposite sides, thus dividing the plane into four equal quadrants, automatically signalled the movement of the animal (counts in 5 min) on the surface of the plane (locomotor activity). Miniature photoelectric cells, in each of the 16 holes, recorded (counts in 5 min) the exploration of the holes (exploratory activity) by the mice.

c Forced swimming test: LOR17 was dissolved in 1:1 saline solution/propylene glycol mixture and subcutaneously administered 25 min before the beginning of the test. Briefly, mice were placed individually into glass cylinders, containing water, and left there for 6 min. The duration of mobility time during the last 4 min was recorded. *** p < 0.001 vs vehicle and LOR17; n = 12 (Newman-Keuls test after ANOVA).

## Discussion and Conclusion

Several studies have proposed that KOPr agonists induce antinociception by activating G protein-mediated cell signaling, including adenylyl cyclase inhibition and early ERK1/2 phosphorylation. Conversely, KOPr-mediated p38MAPK activation contributes to dysphoria and aversion (Bruchas et al., 2007; Ehrich et al., 2015) as well as to sedation and motor incoordination (Bruchas and Roth, 2016). Furthermore, activation of this latter signaling pathway promotes partial sciatic nerve ligation (pSNL)-induced astrocyte proliferation and subsequent hyperalgesia and thermal allodynia (Clayton et al., 2009), and contributes to the modulation of potassium channel heterologous desensitization, possibly leading to antinociceptive tolerance (Xu et al., 2007).

The foremost finding of this study is that LOR17 inhibits adenylyl cyclase to a similar extent as compared to U50,488, but does not significantly recruit β-arrestin 2 at KOPr, displaying a bias factor of 853 toward G protein activation over β-arrestin 2 recruitment. Consistently, LOR17 activates early ERK1/2 phosphorylation in different cell models, whereas contrary to U50,488, it does not activate p38MAPK and does not promote β-arrestin 2-dependent, p38MAPK-mediated astrocyte cell proliferation. Similar to U50,488, LOR17 is effective in animal models of acute nociception. LOR17, more effectively than U50,488, reduces thermal hypersensitivity in a mouse model of oxaliplatin-induced neuropathic pain after both single or repeated s.c. administrations, without altering, in the range of administered doses, motor coordination, locomotor, and exploratory activities and without inducing pro-depressant-like effects.

Thus, LOR17 emerges as a novel, potent and G protein biased KOPr selective agonist with a more favorable *in vitro* and *in vivo* pharmacological profile compared to the classic kappa agonist U50,488. Functionally selective ligands, as LOR17, are indeed valuable research tools to deeply explore and characterize which pharmacological responses are specifically connected to distinct signaling events; thus, allowing to fully understand if and to what extent biased agonism at kappa receptor may be translated into innovative analgesics with reduced adverse effects and low abuse liability.

Notably, functional selectivity at opioid receptors has been intensely investigated in the last decade aiming at identifying more effective and safer analgesics, as many of the unwanted effects determined by MOPr or KOPr agonists are reduced or absent in β-arrestin-2 knock-out mice (Bohn et al., 2000; Raehal et al., 2005; Li et al., 2009; Bruchas and Roth, 2016). Therefore, increasing efforts have been devoted to developing functionally selective opioid agonists displaying a limited activation of arrestin-dependent signaling pathways.

Oliceridine is one of the first MOPr G protein pathway-selective modulators that had undergone phase-IIb clinical trials for the management of moderate to severe acute pain following abdominoplasty (Singla et al., 2017); after that, Oliceridine completed also Phase III clinical trials, but in October 2018 the US FDA decided not to approve it, due to doubts whether the benefits associated with the drug outweighed the risks (Mores et al., 2019). This decision significantly dampened the enthusiasm around biased agonists at opioid receptors (especially at MOPr) as potential improved analgesics. However, FDA decision was taken with 8 votes for rejection and 7 votes for approval; thus highlighting once more that the potential utility of biased agonists at opioid receptors is still highly debated and cannot be completely ruled out at the moment; thus, suggesting that further studies are necessary to fully understand if and how it will be possible to develop novel opioid analgesics more effective and safer than morphine by exploiting functional selectivity at opioid receptors. Moreover, although biased agonism at MOPr has been so far disappointing in terms of getting novel therapeutics into the market, functional selectivity at KOPr is still considered interesting and potentially promising: it is believed, in fact, that the whole kappa opioid field will greatly benefit from future studies aimed at analyzing novel compounds on one hand, and at correlating the degree of signaling bias to particular pharmacological responses on the other (Mores et al., 2019). Within this perspective, we believe that our findings on LOR17 may represent a significant advance.

Regarding functionally selective KOPr agonists, to our knowledge, no any compound has reached clinical trials so far; nevertheless, the finding that KOPr-induced antinociception is G protein dependent, whereas KOPr-mediated dysphoria correlates with p38MAPK-mediated events (Bruchas and Chavkin, 2010), had significantly boosted studies aimed at developing new KOPr agonists with a limited activation of p38MAPK-dependent signaling; thus leading to the identification of different innovative ligands targeting KOPr.

Among these agents, MPCIE, a novel 1-pyrazole methyl ester derivative, elicited KOPr-mediated antinociception without producing sedation, constipation or motor impairment in different models of inflammatory and neuropathic pain (Trevisan et al., 2013); however, the molecular determinants of the MPCIE advantageous profile were not further elucidated.

Zhou and colleagues, on the other hand, have described two classes of biased KOPr agonists potently activating G protein coupling but weakly recruiting β-arrestin-2. The most effective compound of each of these classes (i.e., triazole 1.1 and isoquinolinone 2.1), though, was initially assayed only in the warm-water tail-flick assay, displaying antinociceptive effects similar to U50,488 (Zhou et al., 2013). More recently, triazole 1.1 was shown to induce dose-dependent, KOPr-mediated antinociception and to suppresses chloroquine phosphate–induced scratching responses in C57BL/6 mice without affecting ambulatory behaviors in mice (Brust et al., 2016). Furthermore, antinociception devoid of dysphoria promoted by a KOPr-selective, G-protein-biased agonist, was demonstrated also by other authors (Spetea et al., 2017). On the other hand, White and co-workers have reported that the G protein-biased KOPr agonist RB-64 induces antinociceptive effects in the hot-plate test without impairing rotarod performance or novelty-induced locomotion. Surprisingly, RB-64 is aversive in the conditioned place preference paradigm, albeit it does not involve β-arrestin-2 recruitment at the kappa receptor (White et al., 2015); however, RB-64 did not increase anhedonia in rodents as compared to vehicle in the ICSS paradigm (White et al., 2015). Recently, three KOPr agonists provided with different degrees of functional selectivity toward G protein-dependent signaling were characterized with regard to their neuroendocrine and behavioral effects (Dunn et al., 2018): KOPr-dependent sedative effects elicited by these ligands were symmetrical to their ability to recruit β-arrestin2 at KOPr. Thus, taken together, these data support the hypothesis that downstream of KOPr activation, sedation, coordination impairment and anhedonia may be due to β-arrestin-2 recruitment and aversion possibly related, at least in rodents, to arrestin-independent p38MAPK activation (White et al., 2015).

Our results may represent a significant breakthrough in the understanding of functional selectivity at KOPr and in the development of innovative analgesics.

LOR17, in fact, displayed affinity and selectivity to KOPr similar to the classic kappa agonist U50,488; moreover, both LOR17 and U50,488 significantly inhibited adenylyl cyclase (a G protein-mediated signaling event) in three different cell models expressing hKOPr either exogenously or endogenously, and displayed comparable IC_50_ and E_max_ values across the different cell models.

However, conversely to U50,488, LOR17 did not induce an efficient β-arrestin 2 recruitment at KOPr. The above mentioned differential signaling profile could also be explained in terms of low efficacy, stimulus amplification and receptor reserve (Conibear and Kelly, 2019). Comparing a G protein-dependent output that encompasses high amplification (e.g.: inhibition of cAMP accumulation) to arrestin recruitment at the receptor (which displays little or no amplification) may lead, in fact, to define as “biased” ligands that are not biased at all (Conibear and Kelly, 2019). However, LOR17 inhibited adenylyl cyclase in all the three cell models employed, displaying potency and efficacy similar to U50,488 regardless the significantly reduced KOPr expression levels in U87-MG cells and in human astrocytes as compared to HEK-293/KOPr; thus, supporting the conclusion that LOR17 may be defined as biased toward G protein-mediated signaling.

It has been demonstrated that G protein signaling modulates KOPr-induced antinociception; consistently, both LOR17 and U50,488 produced significant antinociception in two different mouse models of nociceptive pain. These results are in line with the effects elicited by other functionally selective kappa agonists (e.g., triazole 1.1, isoquinolinone 2.1, RB-64) that in comparable paradigms induced antinociception similarly to classic kappa agonists such as U50,488 or U69,593.

In HEK-293/hKOPr cells, in U87-MG cells and in human astrocytes, LOR17 increased ERK1/2 phosphorylation in a KOR-dependent way only after 15 min of exposure, whereas U50,488 triggers early (5–15 min) and late phase (60–120 min) ERK1/2 phosphorylation. This multi-phasic ERK1/2 phosphorylation by classic kappa agonists has already been reported in type-1 immortalized rat cortical astrocytes, and it was hypothesized that early ERK1/2 activation was G protein dependent and that late ERK1/2 phosphorylation could be arrestin-mediated (McLennan et al., 2008). Schattauer and colleagues have shown that acute hKOPr-mediated ERK1/2 activation did not require arrestin recruitment, whereas late phase hKOPr-mediated ERK1/2 phosphorylation was arrestin-dependent (Schattauer et al., 2012). Furthermore, these authors have demonstrated that β-arrestin-2 was necessary for hKOPr-induced p38MAPK phosphorylation (Schattauer et al., 2012), similarly to findings showing that p38MAPK activation downstream of rat KOPr is GRK3- and arrestin-dependent (Bruchas et al., 2006). In HEK-293/hKOPr cells, U87-MG cells and in human astrocytes, LOR17 did not increase p38MAPK phosphorylation even when administered at concentrations up to 100 μM, whereas U50,488 significantly activated it after 30 min of exposure, consistent with a previous report (Schattauer et al., 2012). Moreover, in HEK-293/hKOPr cells and U87-MG cells, LOR17 counteracted U50,488-mediated increase in p38MAPK phosphorylation in a concentration-dependent way. This result is consistent with G-protein-signaling bias for LOR17, but it is also consistent with low efficacy and partial agonism of LOR17 at KOR (Conibear and Kelly, 2019).

Notably, nalfurafine (a moderately selective KOPr analgesic with a low incidence of dysphoric effects used in Japan to treat uremic pruritus) resulted in biased ERK1/2 activation compared to p38MAPK induction in hKOPr-expressing HEK-293 cells and displayed antinociceptive effects in the tail immersion test (Schattauer et al., 2017) as well as in other species and preclinical pain models (Endoh et al., 2000); thus, further sustaining interest in developing KOPr agonists that result in limited p38MAPK phosphorylation (e.g., LOR17). However, it should be noted that nalfurafine was recently demonstrated not to be effective in relieving pain-depressed behaviors (Lazenka et al., 2018); consistently, nalfurafine is not indicated as analgesic drug not even in Japan (where it is marketed as an anti-itch drug). These limited effects displayed by nalfurafine may be related to a G protein signaling bias not high enough to produce a desirable profile of antinociceptive efficacy and safety (Lazenka et al., 2018); thus, pointing at the magnitude of bias factor as another element to be considered when developing new KOPr biased agonists. LOR17 displayed a bias factor of 853 as compared to U50,488, thus emerging as a candidate compound that is worthy of further characterization.

In the present study, we report that U50,488 increased U87-MG astrocytoma and normal human astrocyte cell proliferation in a KOP-dependent, β-arrestin 2- and p38MAPK-mediated fashion, whereas LOR17 did not cause this effect. These findings are relevant considering that U50,488 promotes type II cultured mouse astrocyte cell proliferation (Xu et al., 2007).

In this regard, it is important to remind that the release of the KOPr endogenous agonist dynorphin is significantly increased in at least some animal models of neuropathic pain, including pSNL, partial infraorbital nerve ligation, streptozotocin-induced pain (Jolivalt et al., 2006; Xu et al., 2007; Aita et al., 2010); thus, it potentially involves an initial antinociception due to dynorphin-mediated KOPr activation in neurons followed by neuropathy-induced allodynia and hyperalgesia that may be sustained by dynorphin-evoked KOPr-dependent activation of p38MAPK in astrocytes. Conversely, KOPr agonists inducing G protein signaling without activating p38MAPK (as does LOR17) may promote antinociception by binding to kappa receptors expressed in neurons and may counteract KOPr-dependent p38MAPK activation promoted by endogenous dynorphin by binding to kappa receptor expressed in astrocytes; thus reducing, or possibly preventing, endogenous dynorphin’s detrimental effects. Within this frame, our findings showing that LOR17 counteracts in a concentration-dependent fashion the increase in astrocyte cell proliferation induced by U50,488 are relevant, as they support the hypothesis that LOR17 is indeed able to counteract arrestin-mediated outcomes triggered by classic KOPr agonists.

LOR17 significantly reduced thermal hypersensitivity after both acute and repeated subcutaneous injections in a mouse model of oxaliplatin-induced neuropathic pain; thus, suggesting that LOR17 is effectively absorbed following peripheral administration and does not cause tolerance after repeated administrations. Conversely, U50,488 significantly reduced reduces thermal hypersensitivity only when administered at 20 mg/kg. This functional selectivity displayed *in vivo* by LOR17, compared to U50,488, may be related to the lack of p38MAPK activation by the former agent. No information has been reported on endogenous dynorphin release in the oxaliplatin-induced neuropathy paradigm adopted. However, this neuropathy model has been characterized with regard to peripheral and central glial activation, showing initial microglial activation followed by a more prolonged astrocytic reactive state in the spinal cord and in different brain regions related to nociception (Di Cesare Mannelli et al., 2013). Thus, these data further support the suggestion that the noteworthy effectiveness of LOR17 in counteracting oxaliplatin-induced neuropathic behaviors may rely on the lack of p38MAPK-dependent glia activation. However, additional studies will be necessary to further characterize this aspect *in vivo* and to assess any contribution of endogenous dynorphin to the neuropathic symptoms triggered by oxaliplatin administration. Nonetheless, LOR17 effectiveness in reducing chemotherapy-induced thermal hypersensitivity may be clinically relevant, considering that oxaliplatin is a widely used anticancer drug and its neurotoxicity is long-lasting (Tofthagen et al., 2013) and represents one of its major dose-limiting side effects (Kannarkat et al., 2007).

To characterize any unwanted effect elicited by LOR17 on motor coordination, locomotor, and exploratory activity and mood, specific behavioral tests were carried out by comparing vehicle to 10 mg/kg LOR17; this dose was selected as it is the lowest dose at which we observed the maximal reduction in oxaliplatin-induced thermal hypersensitivity.

LOR17 (10 mg/kg) does not significantly alter motor coordination, locomotor and exploratory activities and does not cause pro-depressant-like effects. Conversely, U50,488 (10 mg/kg) significantly reduced rotarod performance and decreased the mobility time in the forced-swim test. These findings are in agreement with previous data showing that 10 mg/kg U50,488 (Ehrich et al., 2015) or 10 mg/kg U69,593 (Mague et al., 2003) significantly reduced locomotor activity compared to vehicle. It has been reported that U69,593 (0.1–10 mg/kg) dose-dependently induces pro-depressant-like behaviors (Mague et al., 2003) and 1 mg/kg U69,593 significantly decreases novelty-induced locomotion and determines anhedonia-like effects (White et al., 2015). Thus, providing further support for the LOR17 advantageous pharmacological profile. Furthermore, the fact that LOR17 could block *in vitro* ßarrestin-mediated effects of U50488, is suggestive that this new KOPr agonist may be able to counteract as well U50,488-induced effects on rotarod performance and forced-swim behaviors. Further studies will be carried out to assess whether, and to what extent, LOR17 is able to counteract β arrestin-dependent effects induced by U50,488 *in vivo*; if confirmed, this functional antagonism will add further value to the favorable pharmacological profile displayed by LOR17.

Given that LOR17 is a peptide, the above mentioned favorable profile could be also related to a reduced distribution to the central nervous system. It has been reported, in fact, that activation of peripheral KOPr by D-amino acid peptide agonists determined a significant antinociception in the writhing test in mice, at doses lower than those required to alter rotarod performance in the same animals (Vanderah et al., 2008); moreover, these peptide agonists determined a significant antinociception in the warm water tail withdrawal test only when administered at doses higher than those that were effective in the writhing test (Vanderah et al., 2008). Thus, further confirming that the peptide agonists reported in the above mentioned study were mainly peripherally active (the tail withdrawal test was included as an experimental paradigm for centrally-mediated antinociception) (Vanderah et al., 2008). Notably, we show that LOR17 determine a significant antinociception both in the acetic acid-induced writhing assay and in the warm water tail-withdrawal assay, when administered in the same range of doses. Thus, suggesting that LOR17 may distribute to the central nervous system. Furthermore, in the study mentioned above, Vanderah and colleagues employed the ratio between potency in tail immersion test and potency in writhing test as peripheral selectivity index: according to this analysis, enadoline (brain penetrating KOPr peptide agonist) and asimadoline (peripherally selective KOPr peptide agonist) displayed a peripheral selectivity index equal to 4 and 13, respectively, whereas the innovative peptide agonists described in that study showed peripheral selectivity indexes equal to 242 and 1429 (Vanderah et al., 2008). By applying the same analysis to U50,488 and LOR17 potencies in tail immersion and writhing assays, a peripheral selectivity index of 1.2 for U50,488 and 1.75 for LOR17 can be determined. These findings further support that LOR17 may distribute to the central nervous system. Nonetheless, further studies will be carried out to fully characterize LOR17 pharmacokinetics.

To conclude, we believe that the findings reported in this study set a sound foundation to support that KOPr selective agonist LOR17 may be a useful research tool to further characterize the functional selectivity of KOPr agonists, both *in vitro* and *in vivo*, and might represent interesting lead compound to develop more effective and safer analgesics. Future studies aimed at expanding LOR17 *in vivo* characterization by means of multiple experimental paradigms and different species will be necessary, however, to fully confirm its selectivity to KOPr *in vivo* and to assess to what extent the noteworthy premises observed here will be fulfilled.

## Data Availability Statement

The datasets generated for this study are available on request to the corresponding author.

## Ethics Statement

The animal study was reviewed and approved by Animal Care and Use Committee of the University of Bologna (Prot. n. 29-IX/9, 25th July 2012); Animal Subjects Review Board of the University of Florence.

## Author Contributions

AB conceived the study, designed the experiments, carried out the majority of the work, analyzed the data and contributed to write the manuscript. LDCM and LM contributed to design the experiments, performed *in vivo* experiments, and contributed to analyze the data and to write the manuscript. MB and GV contributed to design and perform *in vitro* experiments. RM and LG designed, synthesized, and analyzed the chemical compounds. CG, LG, and SS designed the study and wrote the manuscript. All the authors revised and approved the final version of the manuscript.

## Funding

This work was supported by grants from the University of Bologna FARB FFBO 125290, RFO 2014, RFO 2015, RFO 2016 to SS and AB; by grants from MIUR (PRIN 2010 and PRIN 2015) to SS and LG; by a research grant from Fondazione Veronesi-Milano (Proj. Tryptoids) to SS, AB, LG, RDM; by research grants from University of Florence and Italian Ministery of University, Research and Instruction to CG and LDCM.

## Conflict of Interest

The authors declare that the research was conducted in the absence of any commercial or financial relationships that could be construed as a potential conflict of interest.

## Supplementary Material

Data Sheet 1 Raw images of each representative western blot; images are displayed in the same order as in the main article.

## Abbreviations

ABM, astrocyte basal medium; BSA, bovine serum albumin; CIPN, chemotherapy-induced peripheral neuropathy; CL, control latency; DAMGO, [D-Ala2, N-MePhe4, Gly-ol]-enkephalin; DIPEA, N,N-diisopropylethylamine; DMF, dimethylformamide; DPDPE, [D-Pen2,D-Pen5]enkephalin; GPCR, G-protein coupled receptor; hDOPr, human delta opioid receptor; hKOPr, human kappa opioid receptor; hMOPr, human mu opioid receptor; HOBt, hydroxybenzotriazole; i.p., intraperitoneal; IASP, International Association for the Study of Pain; IL-1b, interleukin-1 b; KOPr, kappa opioid receptor; MOPr, mu opioid receptor; MW, micro waves; NMR; nuclear magnetic resonance; norBNI, norbinaltorphimine; pIONL, partial infraorbital nerve ligation; pSNL, partial sciatic nerve ligation; RP-HPLC, reversed-phase high-performance liquid chromatography; s.c., subcutaneous; STZ, streptozotocin; TL, test latency.
