# Supplementary figures and images for: Functional Selectivity and Antinociceptive Effects of a Novel KOPr Agonist
Source: Front Pharmacol. 2020 Mar 5;11:188. doi: 10.3389/fphar.2020.00188 (PMC7066533; doi:10.3389/fphar.2020.00188)

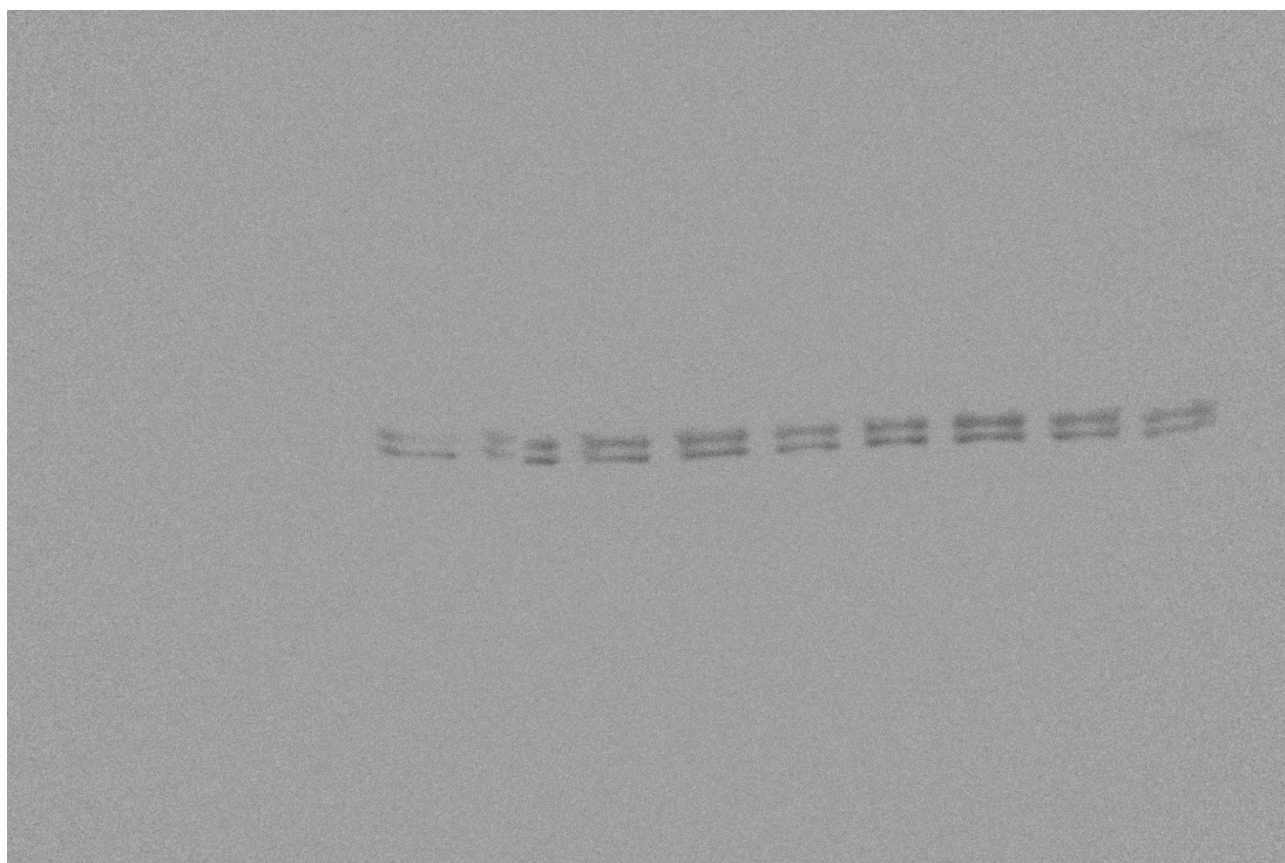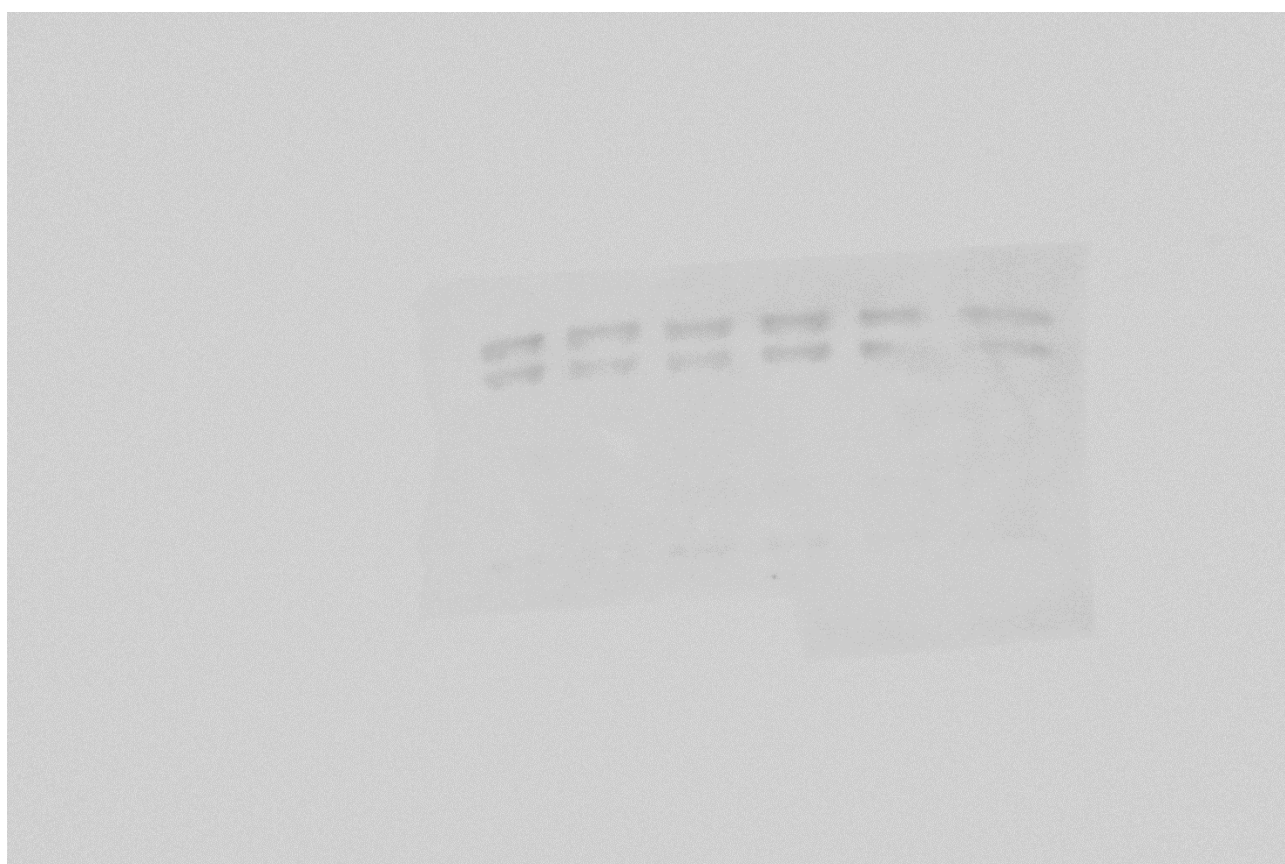

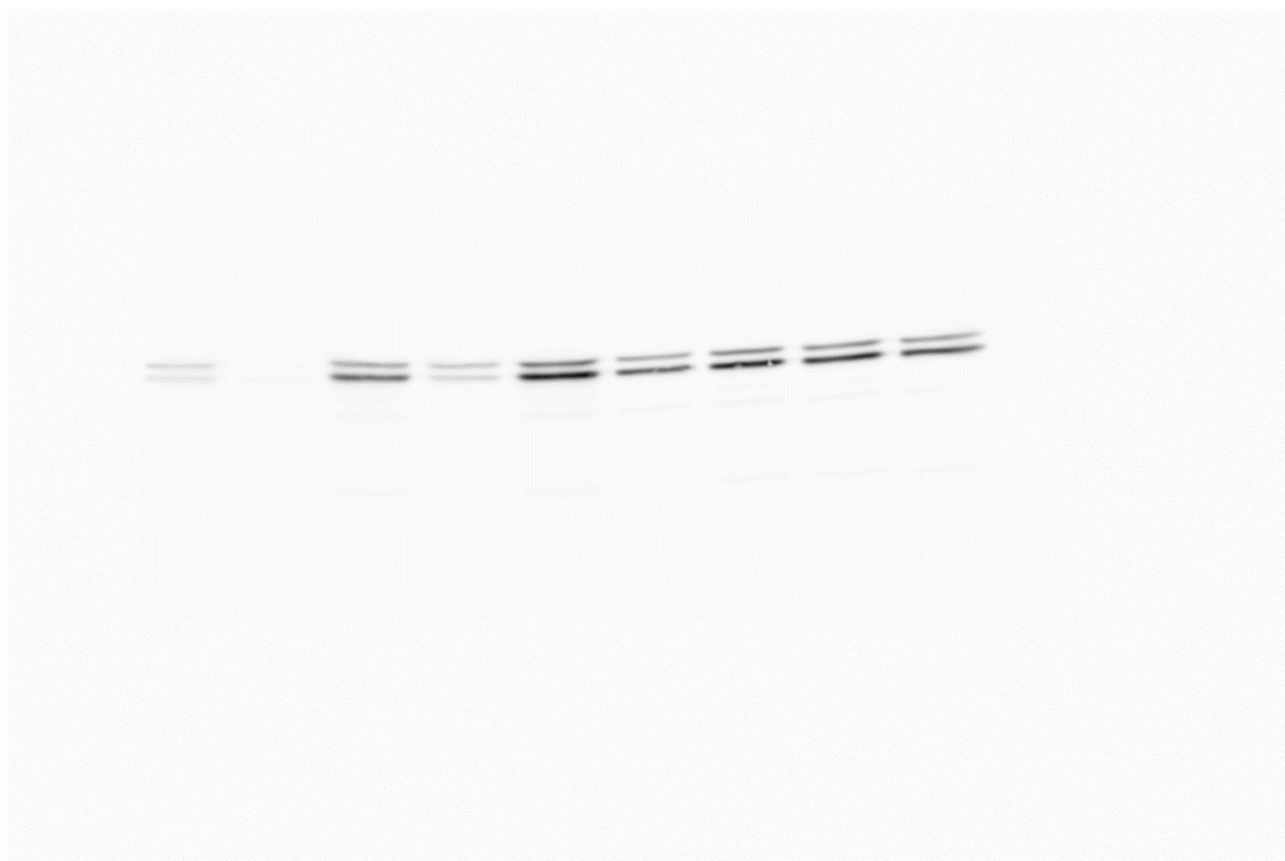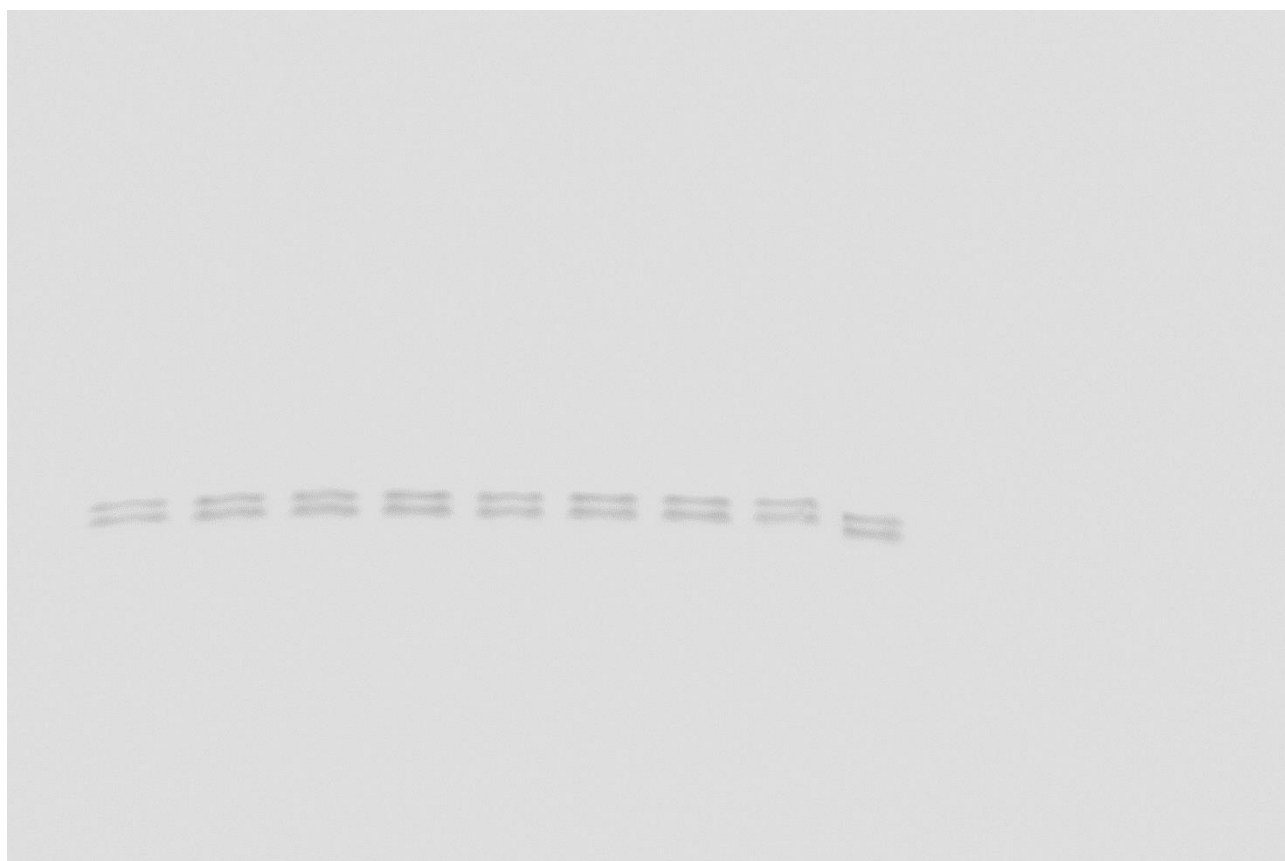

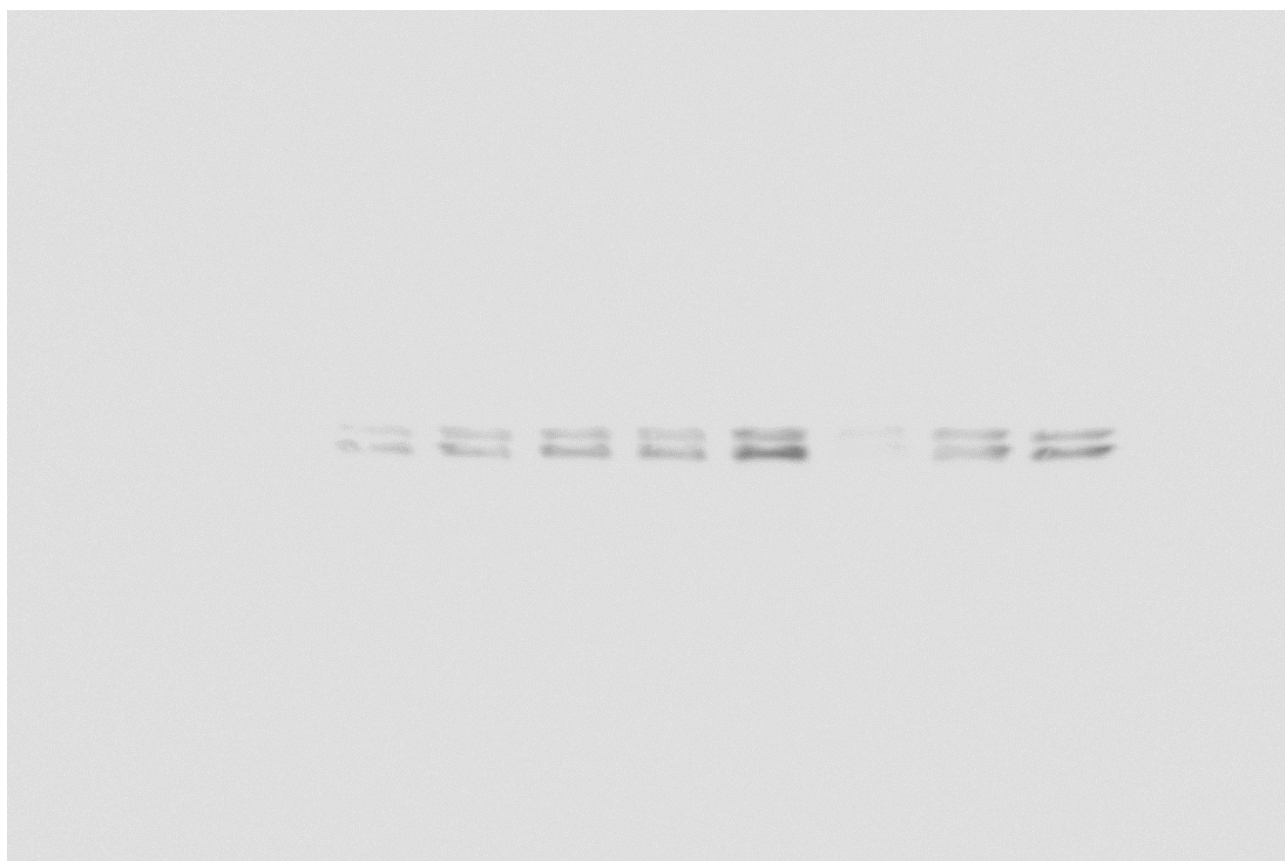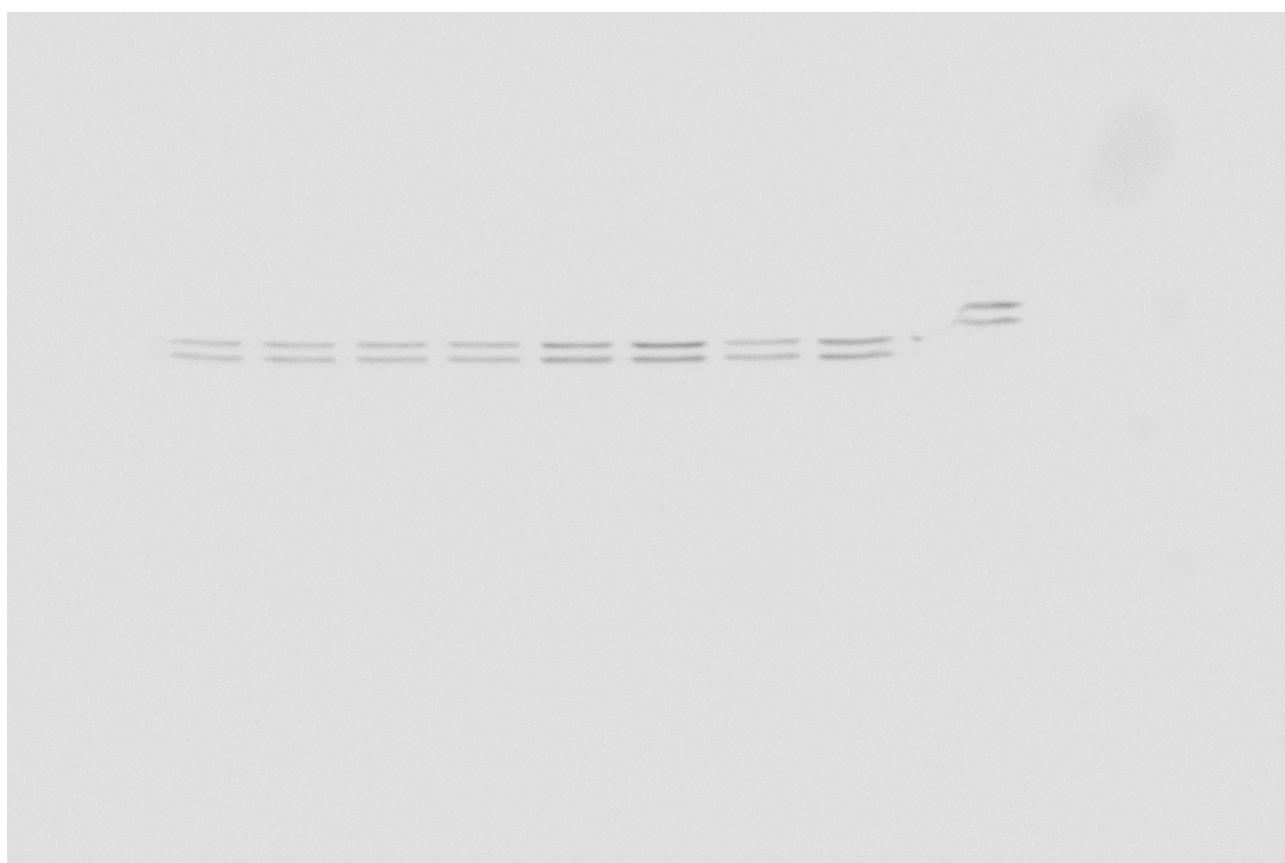

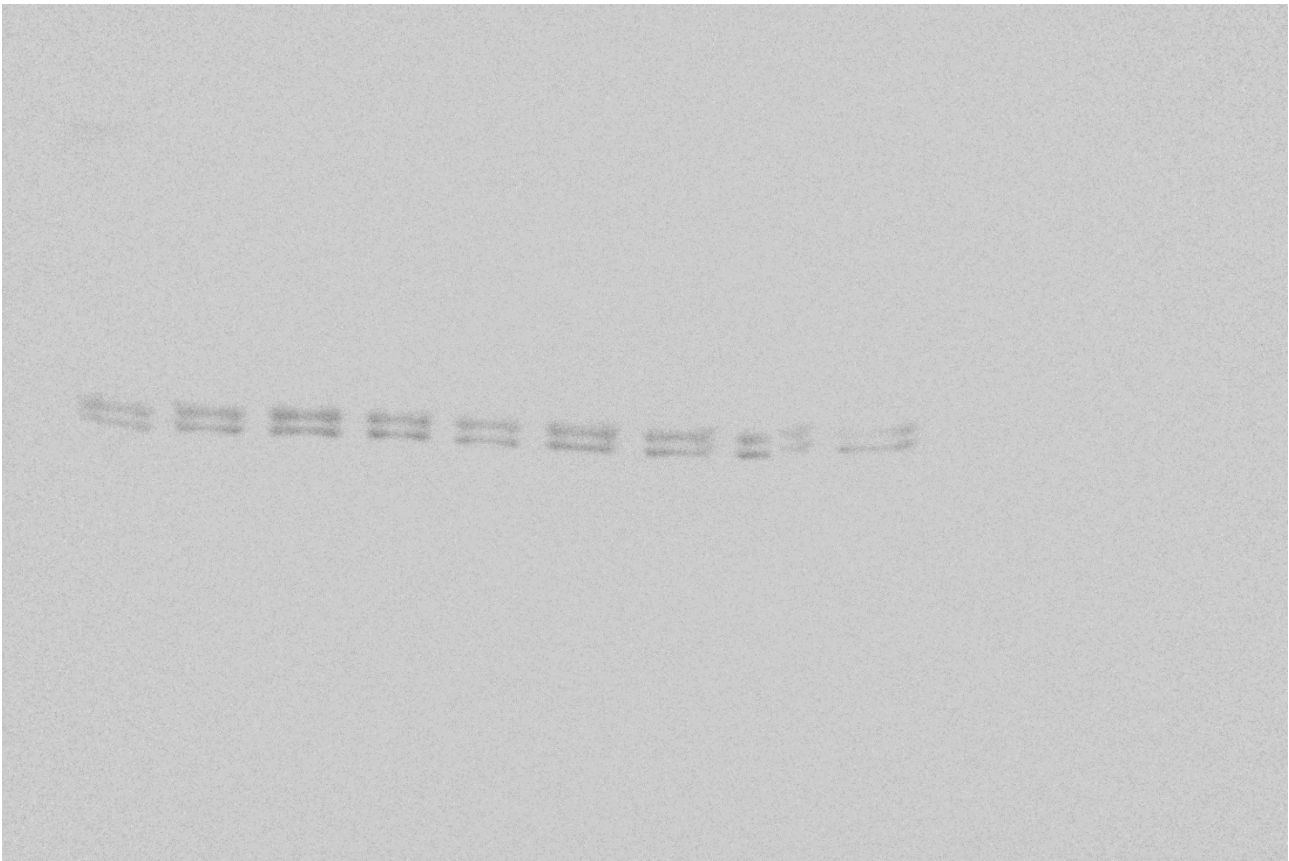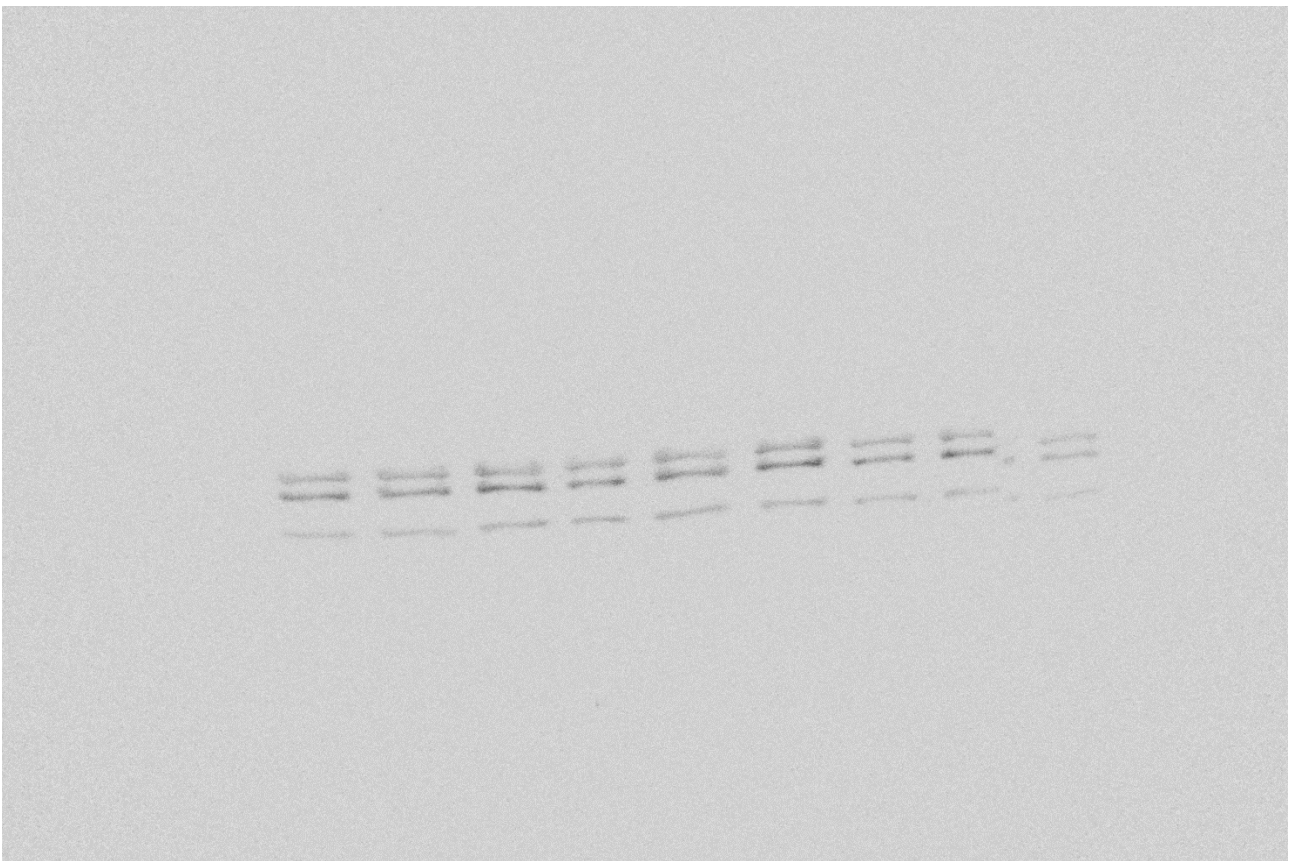

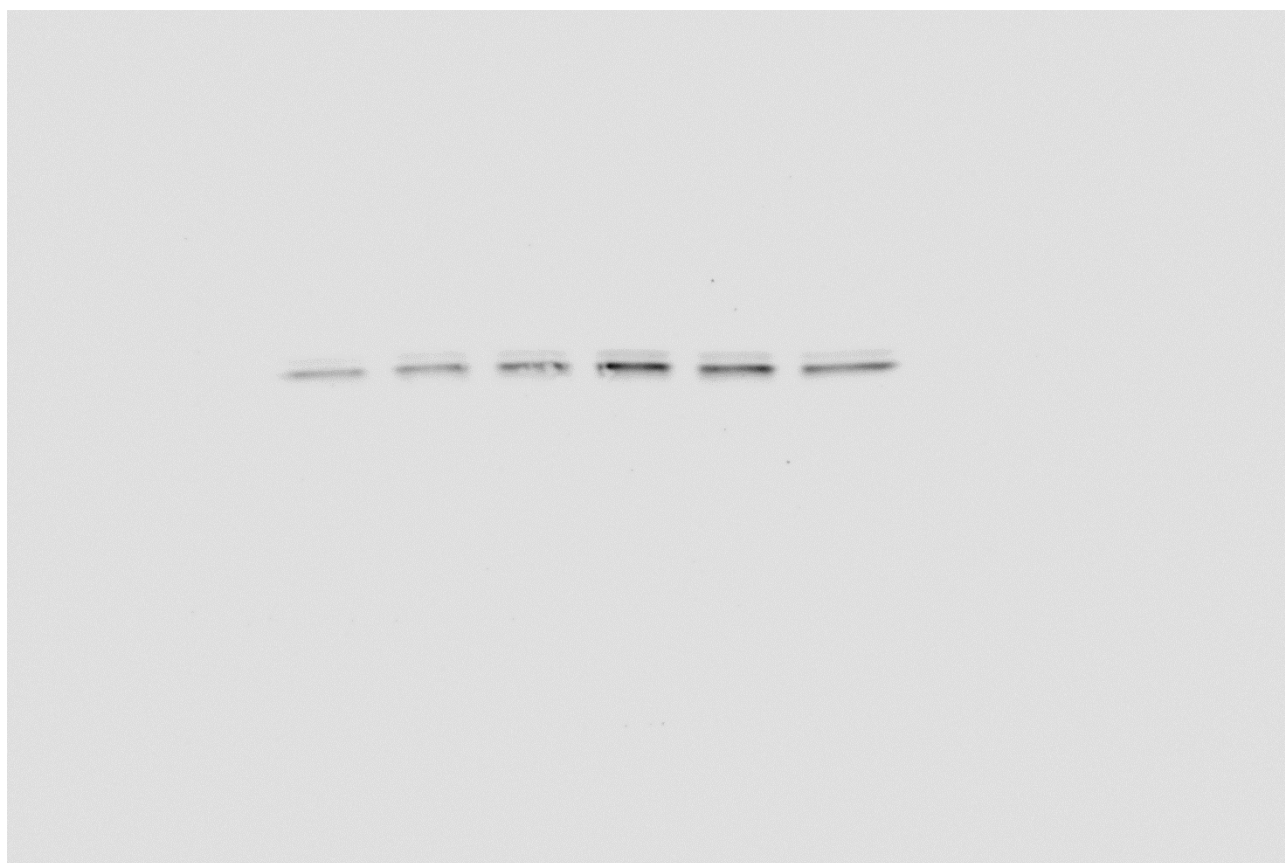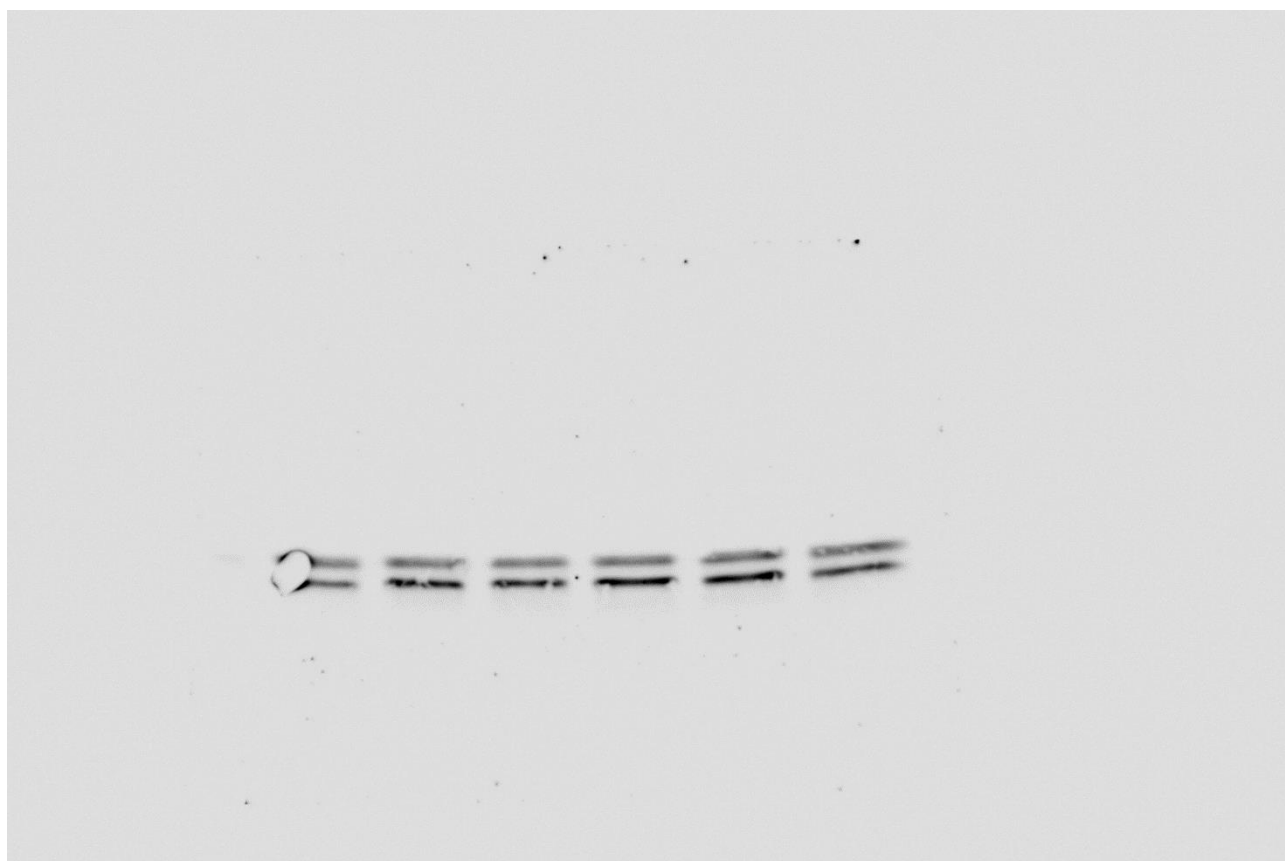

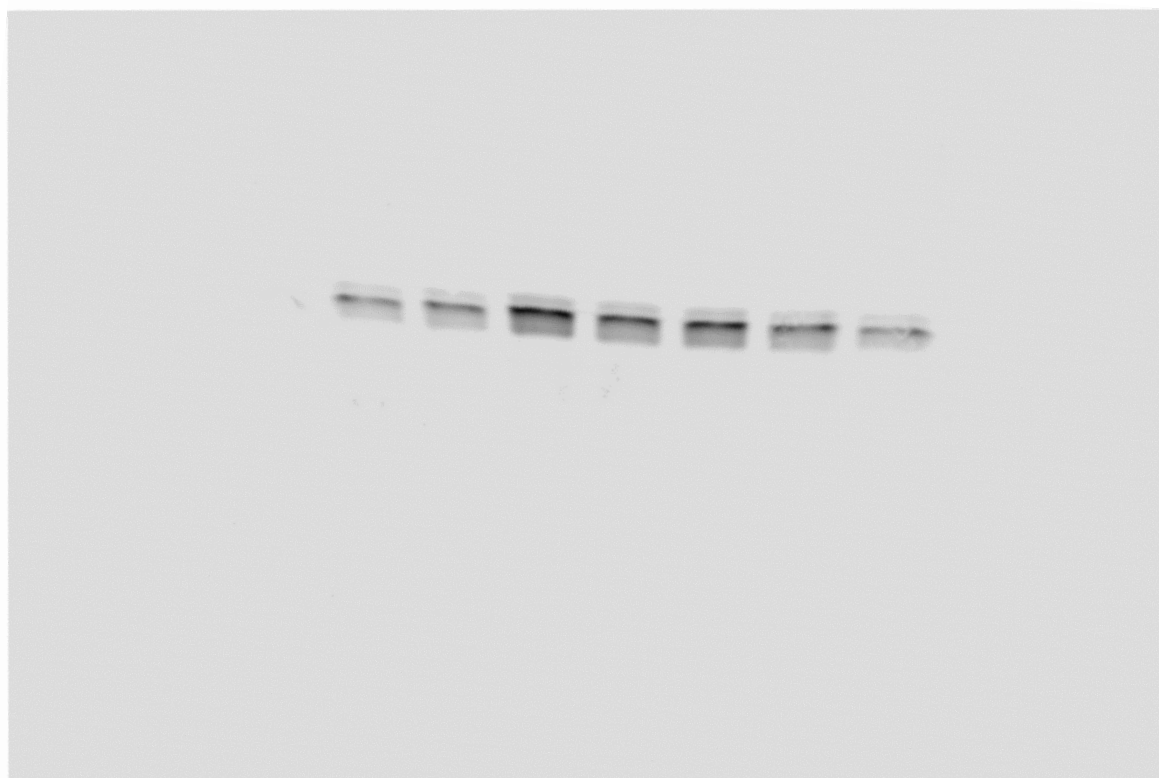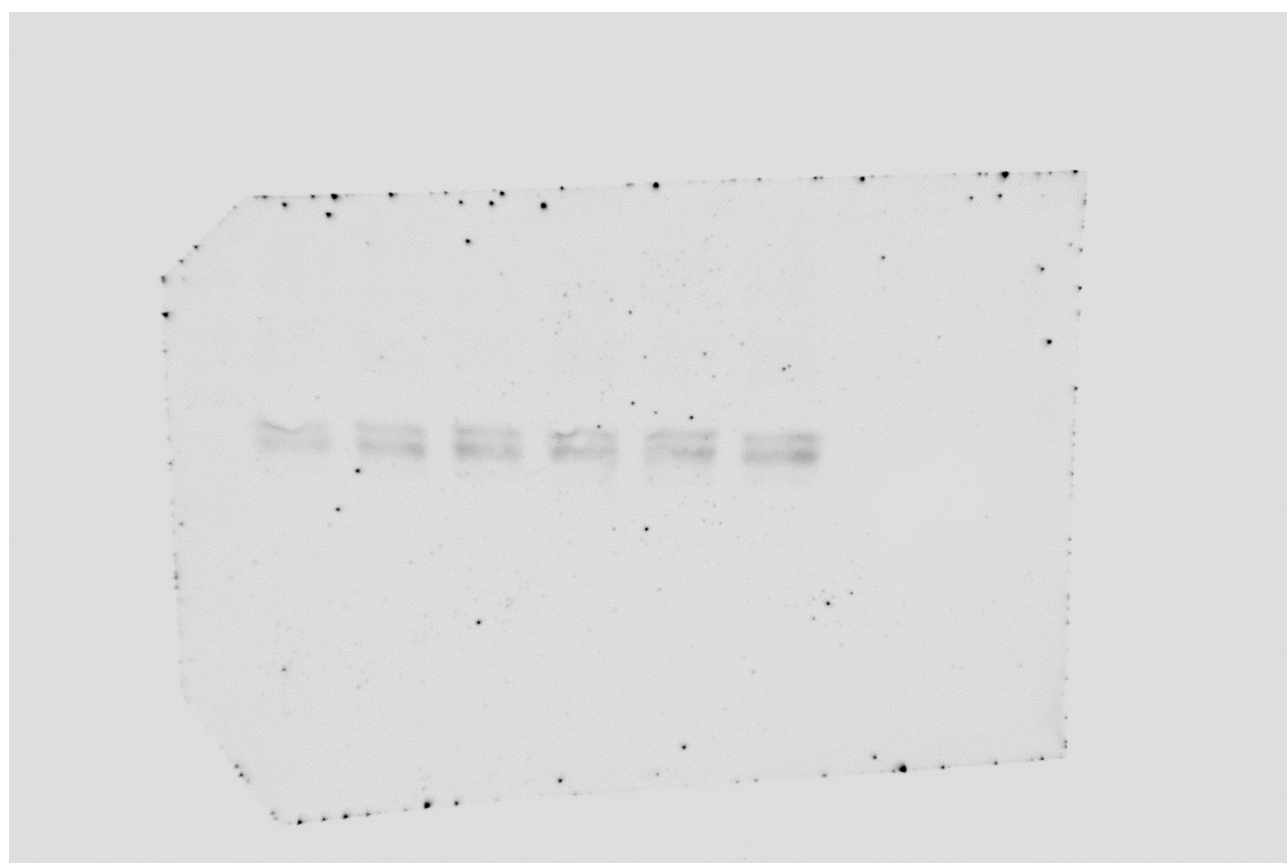

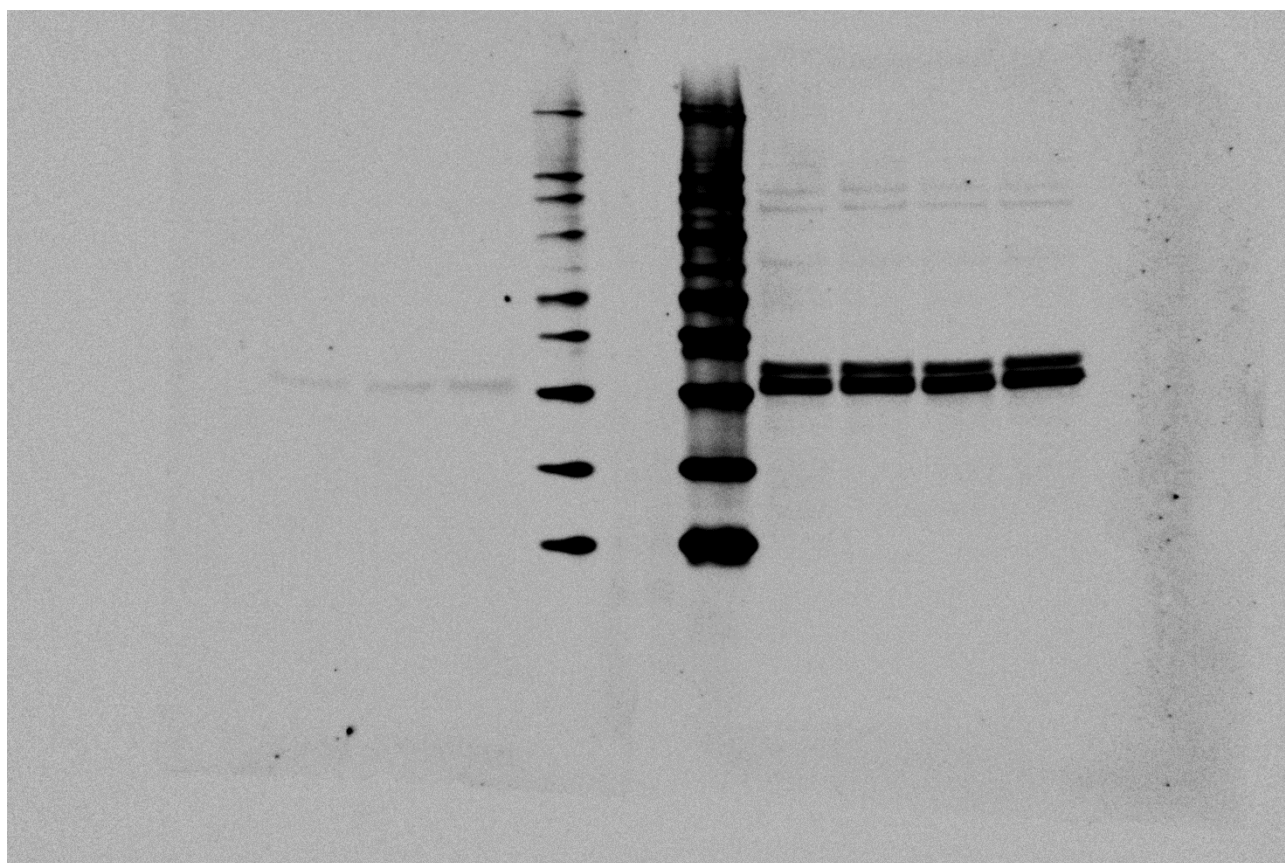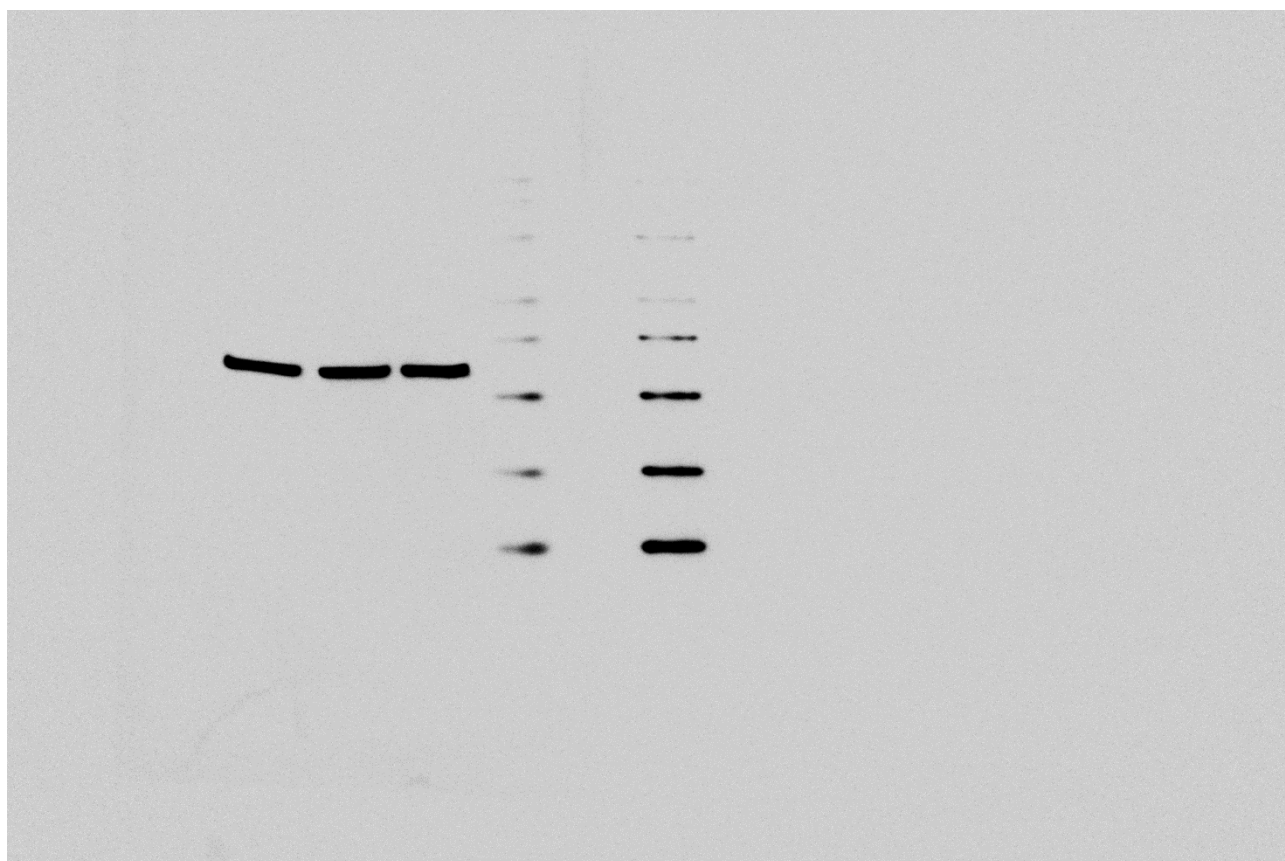

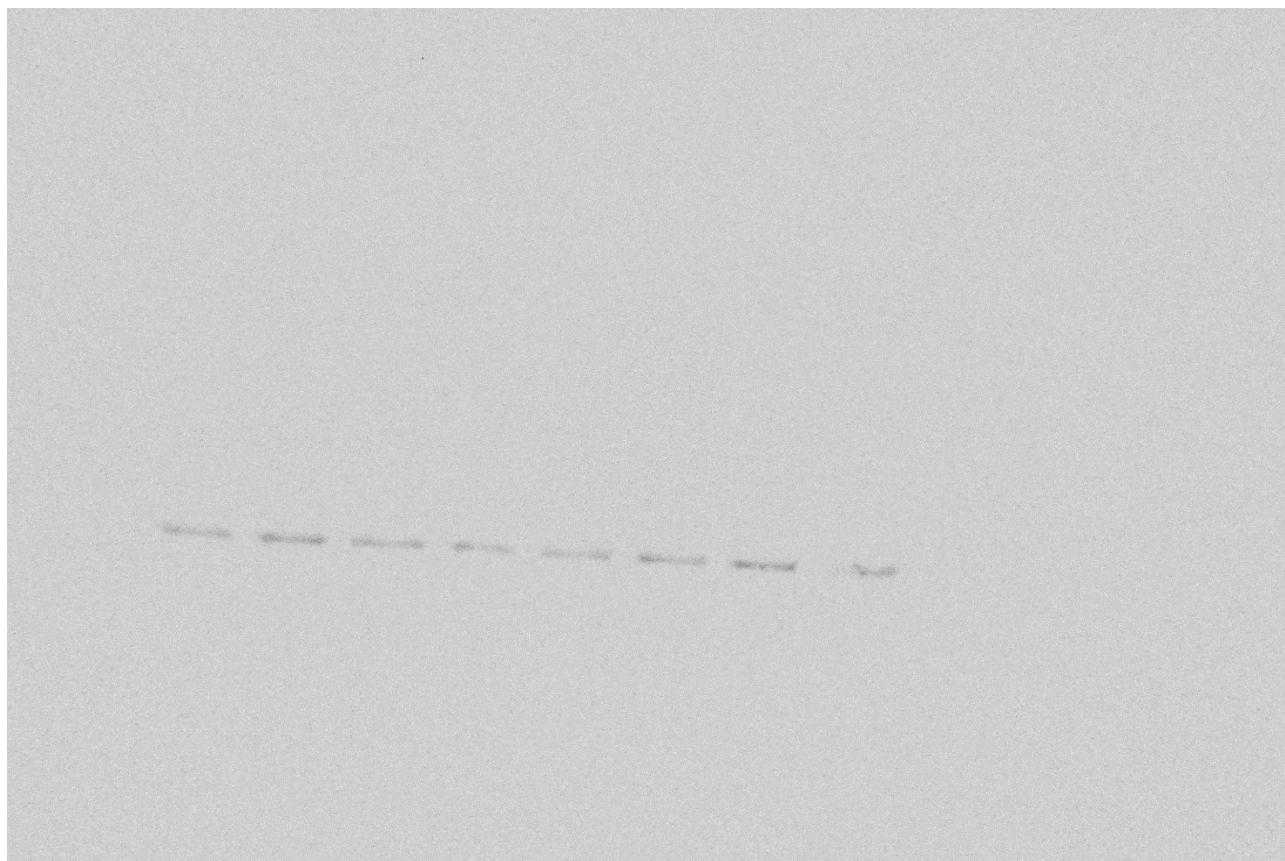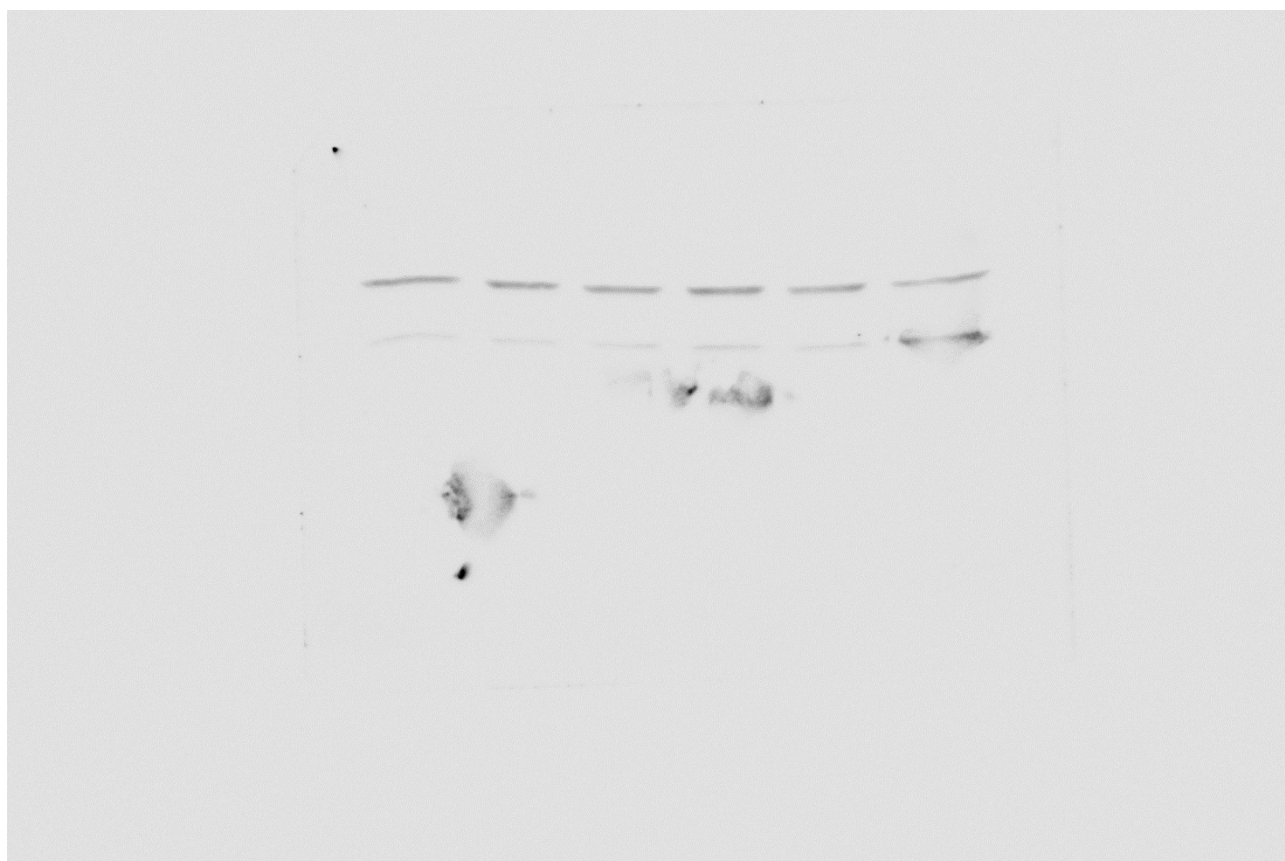

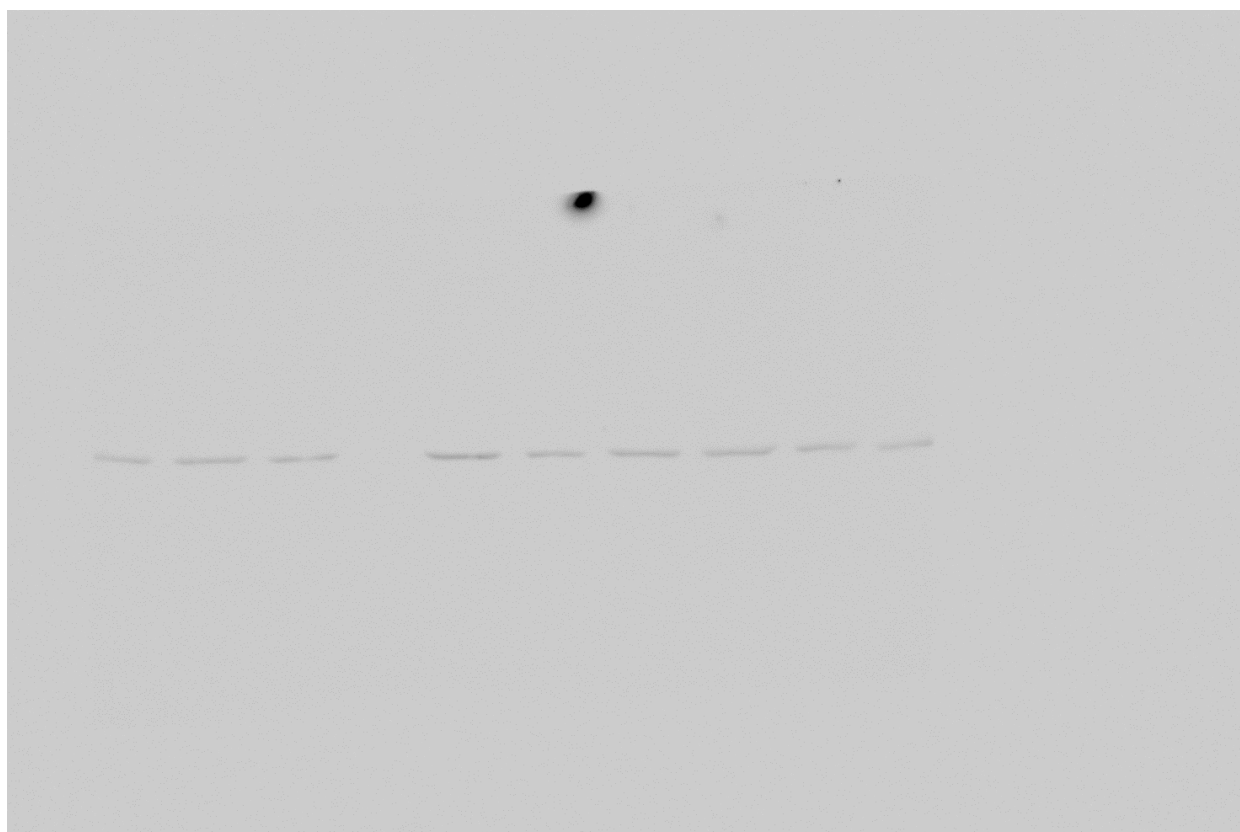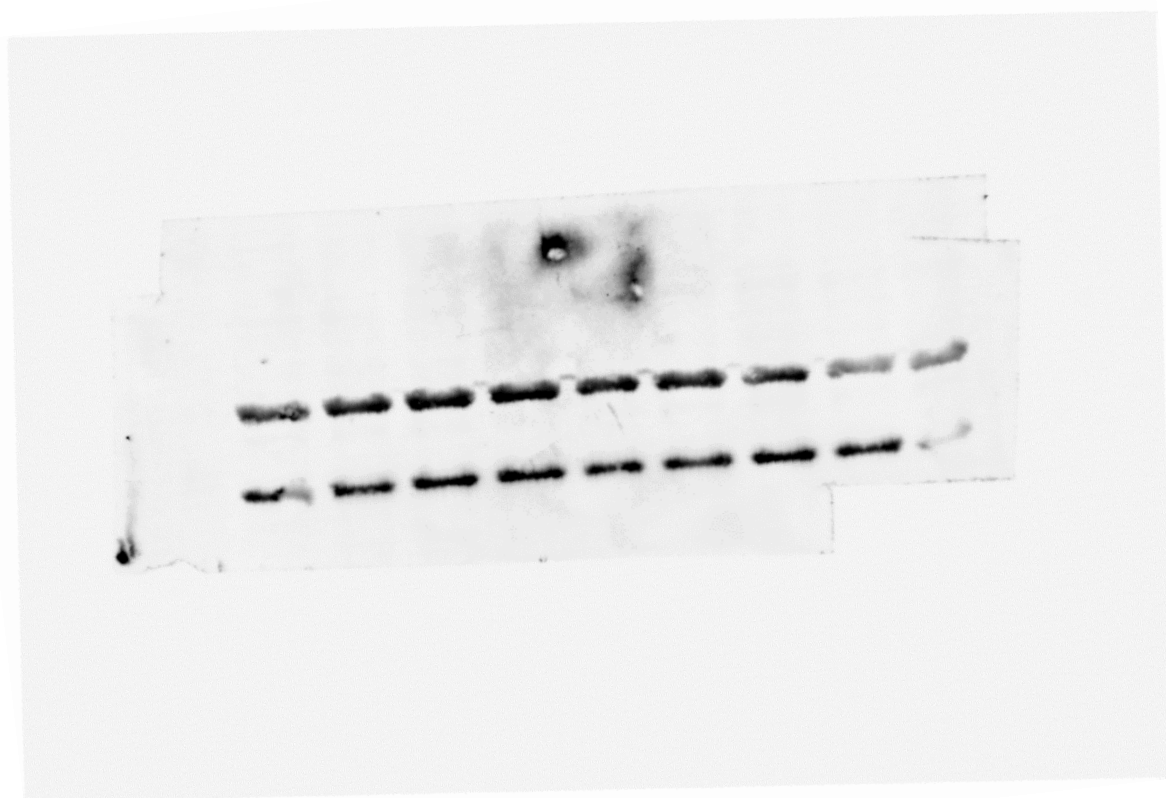

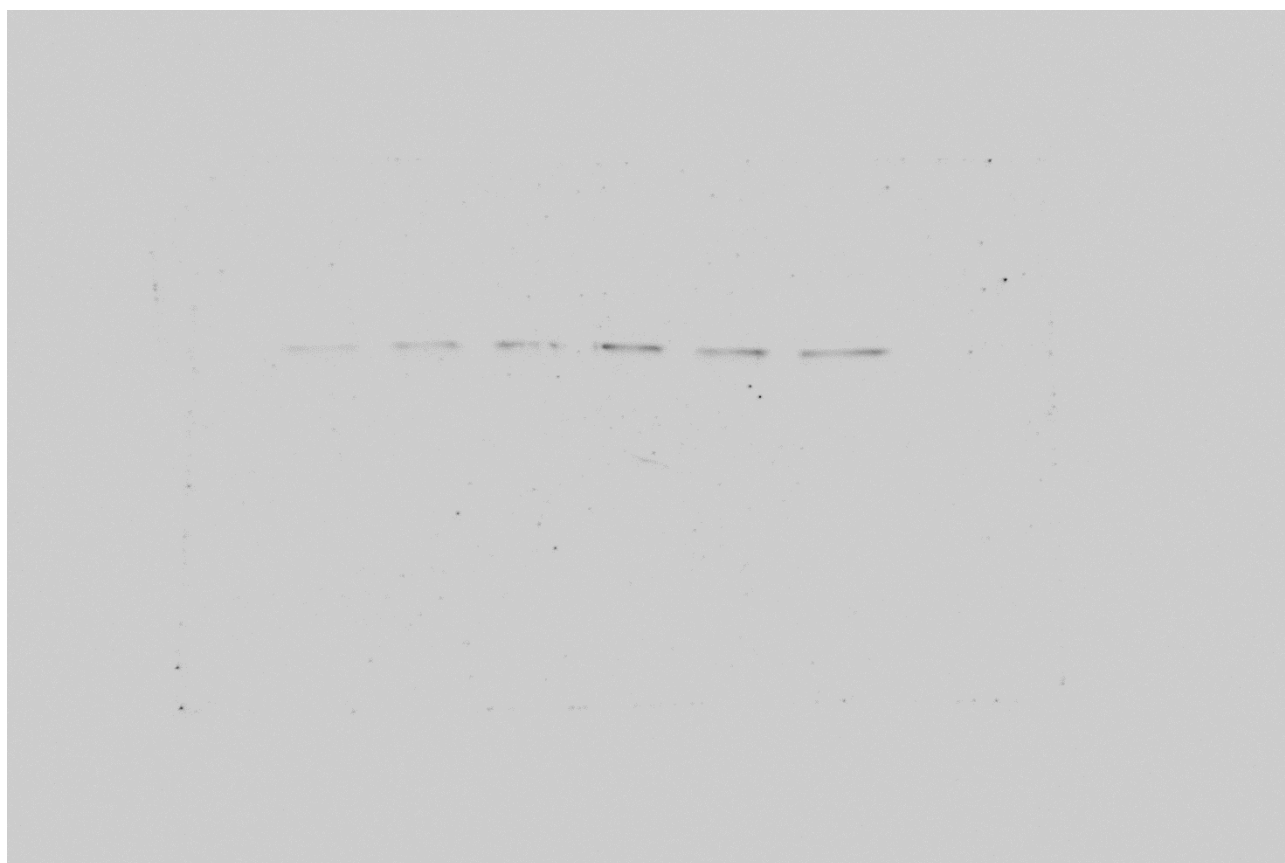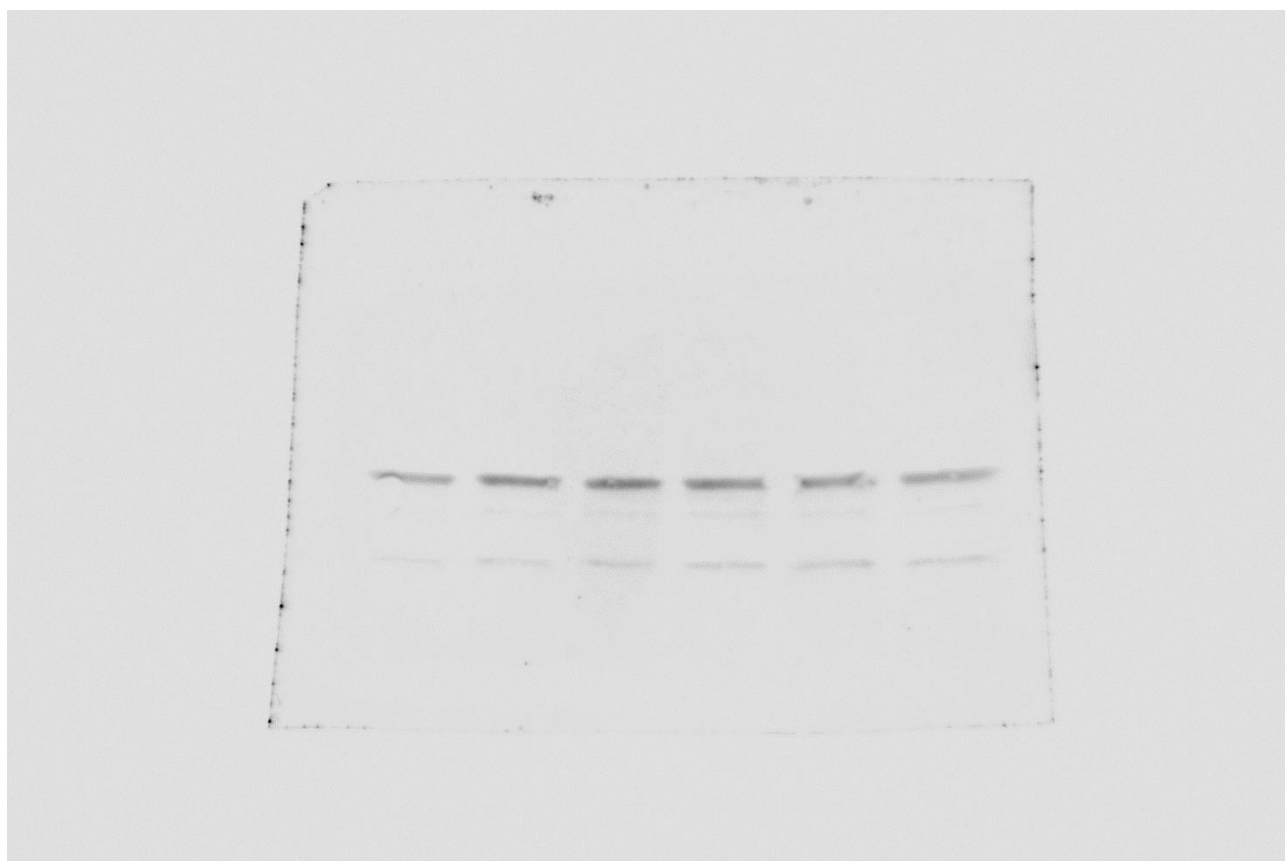

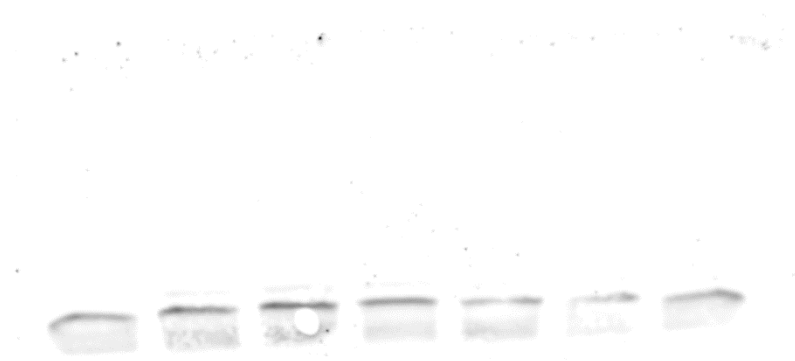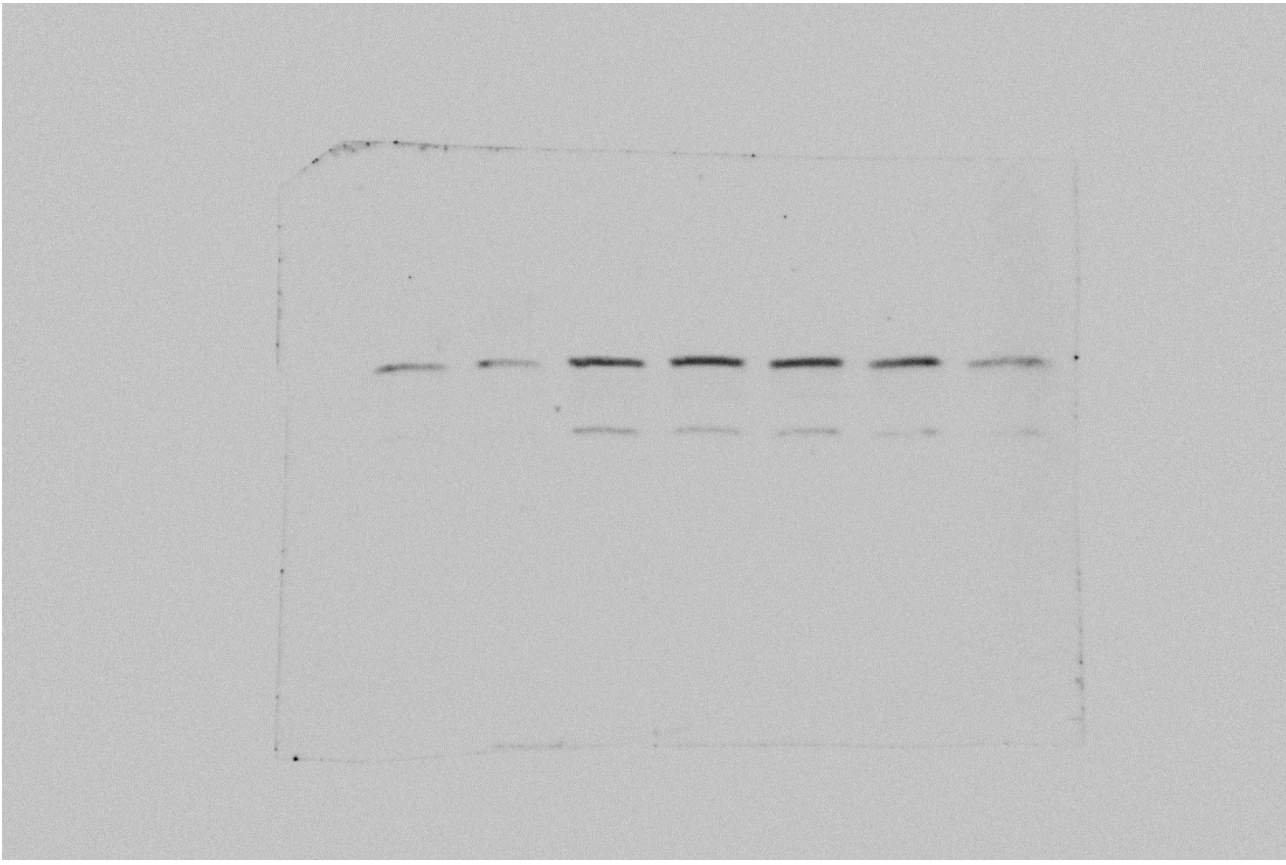

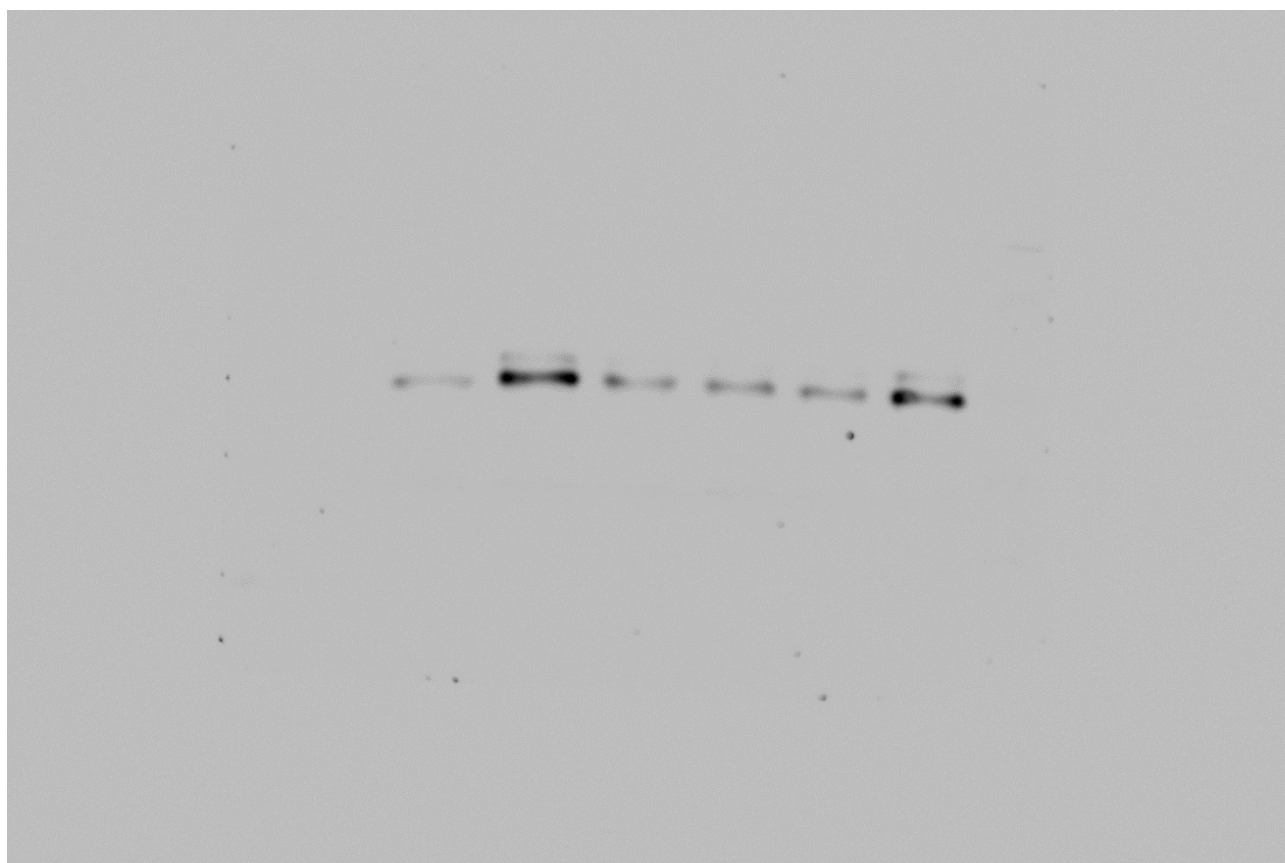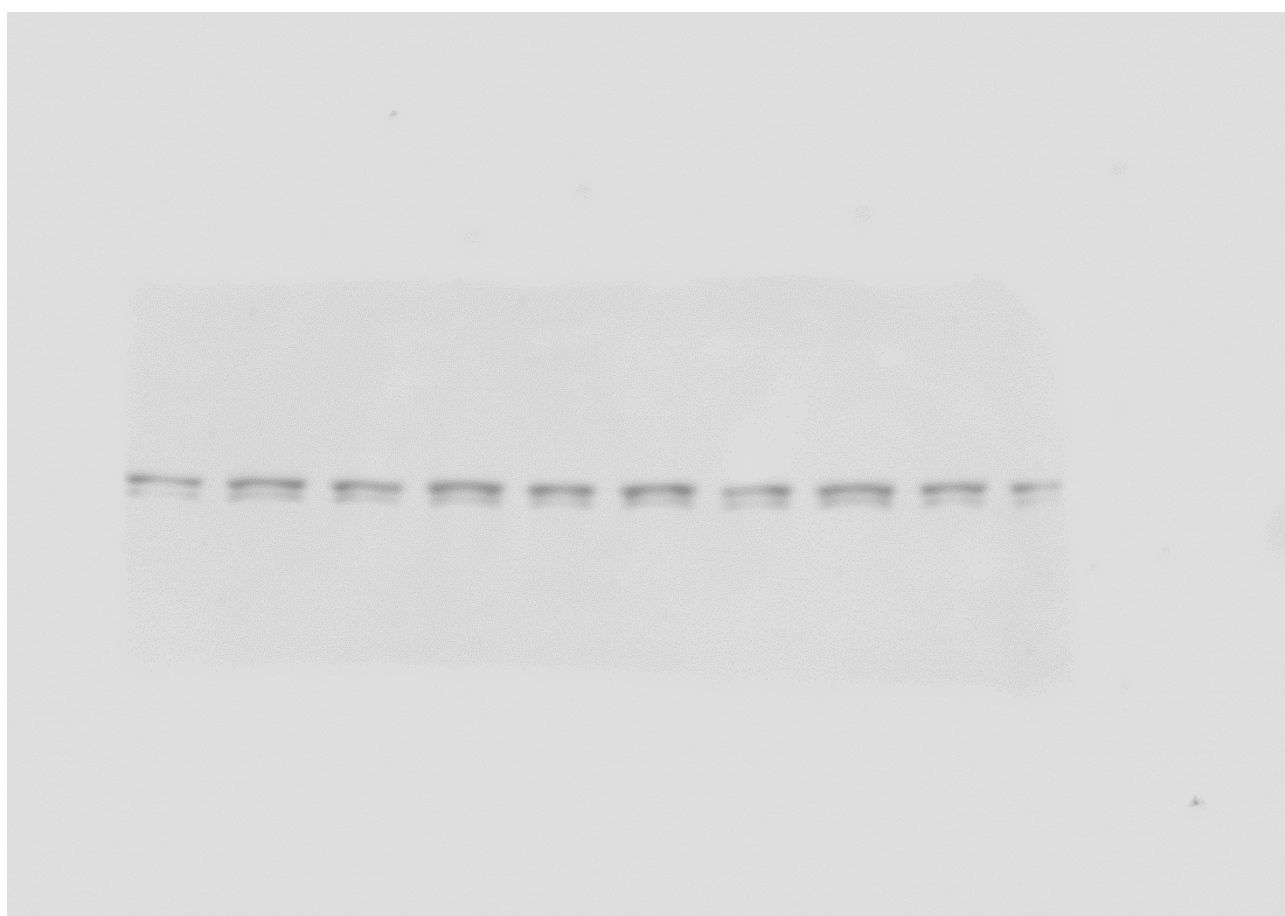

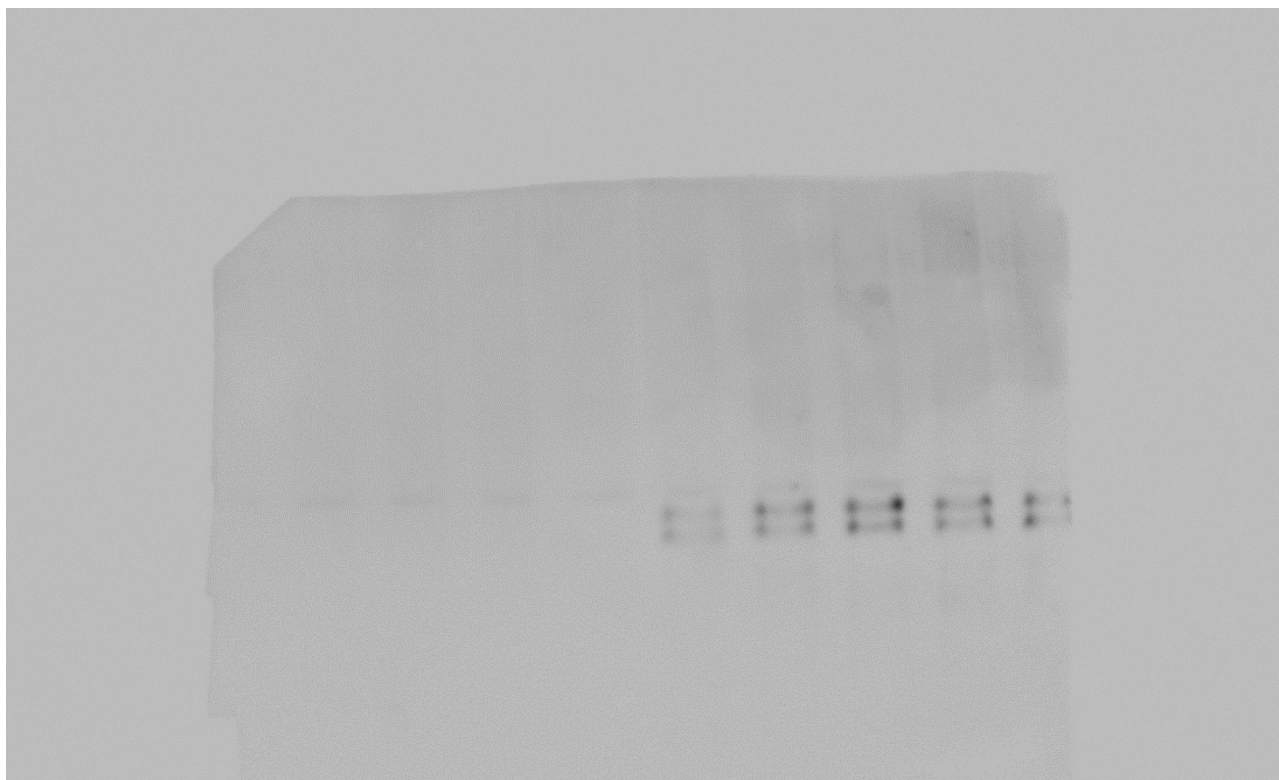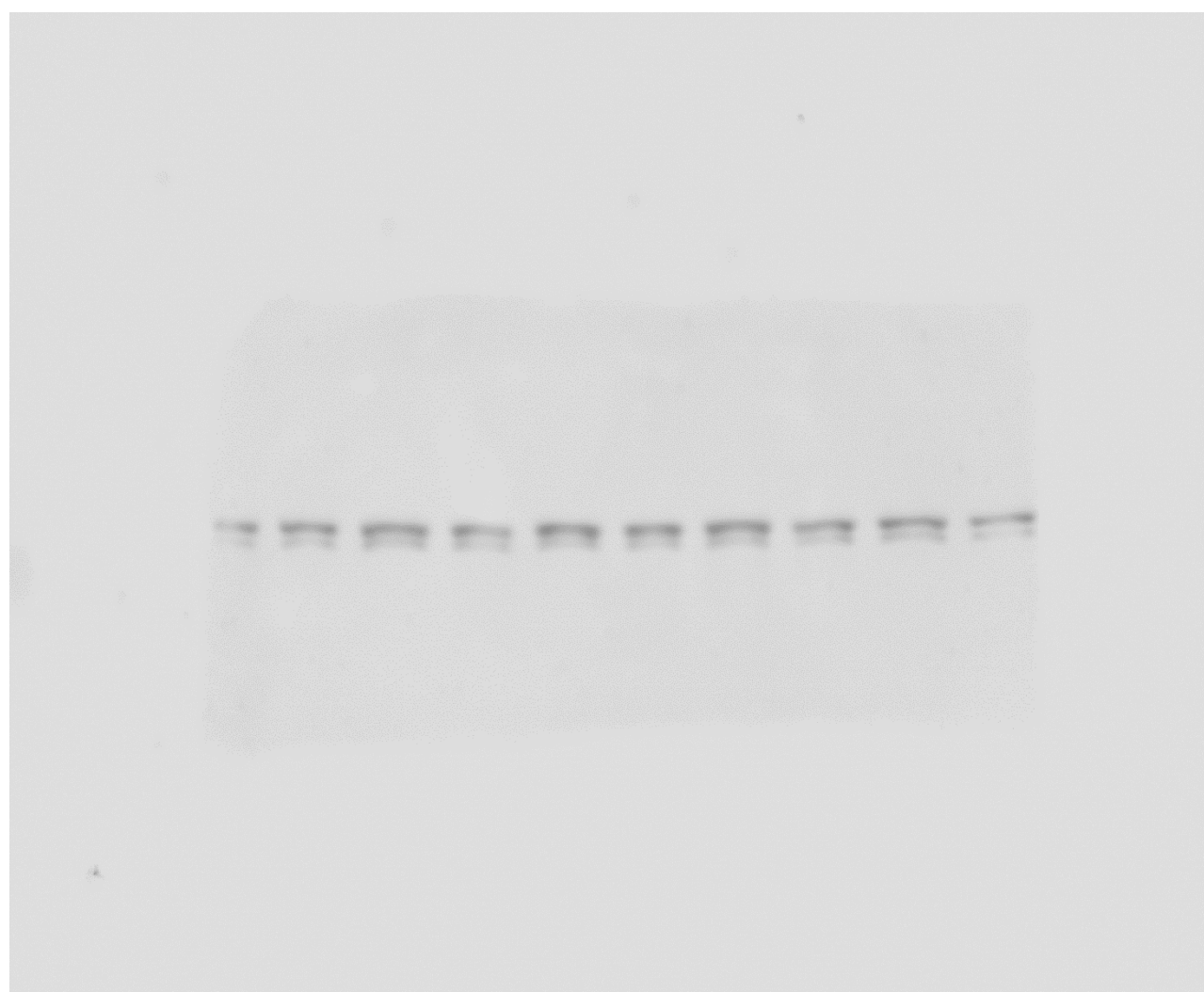

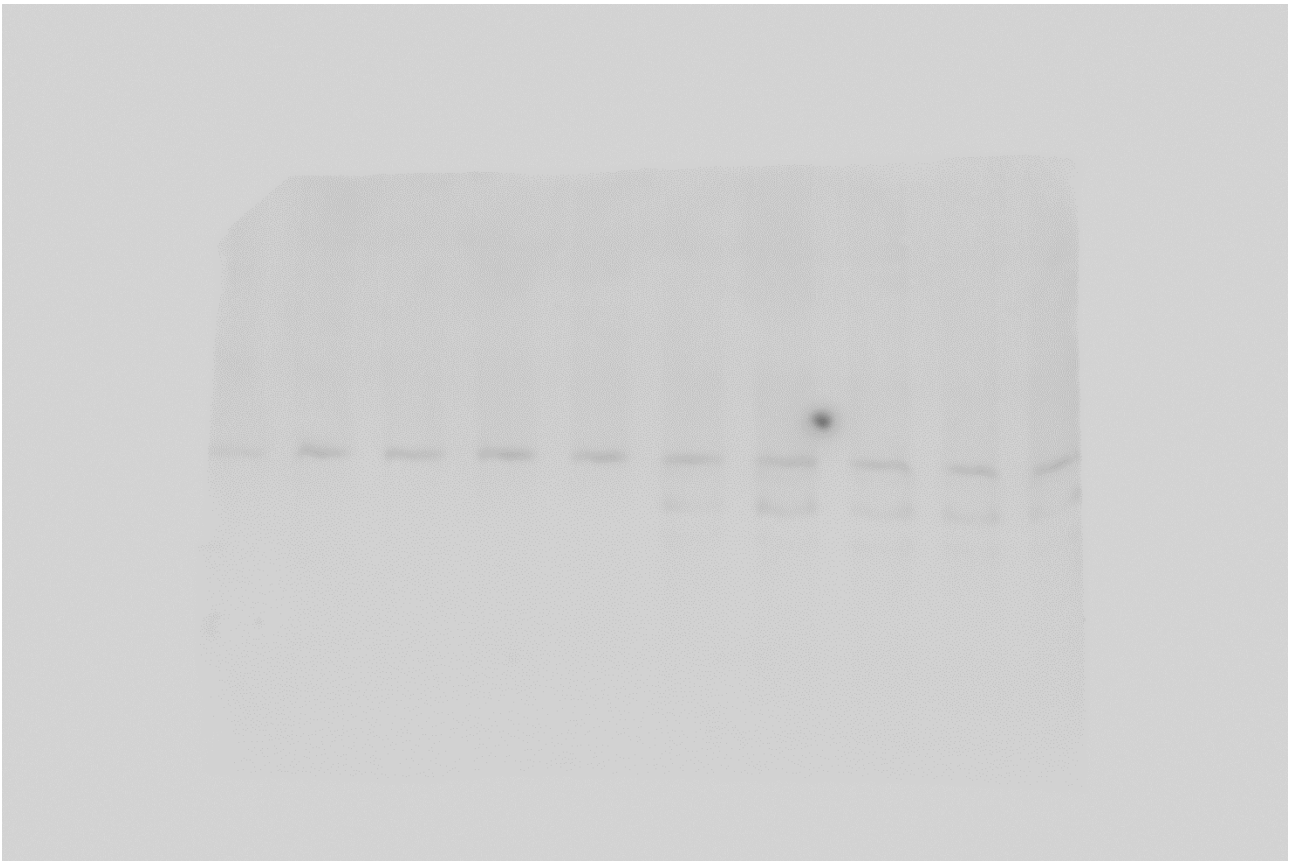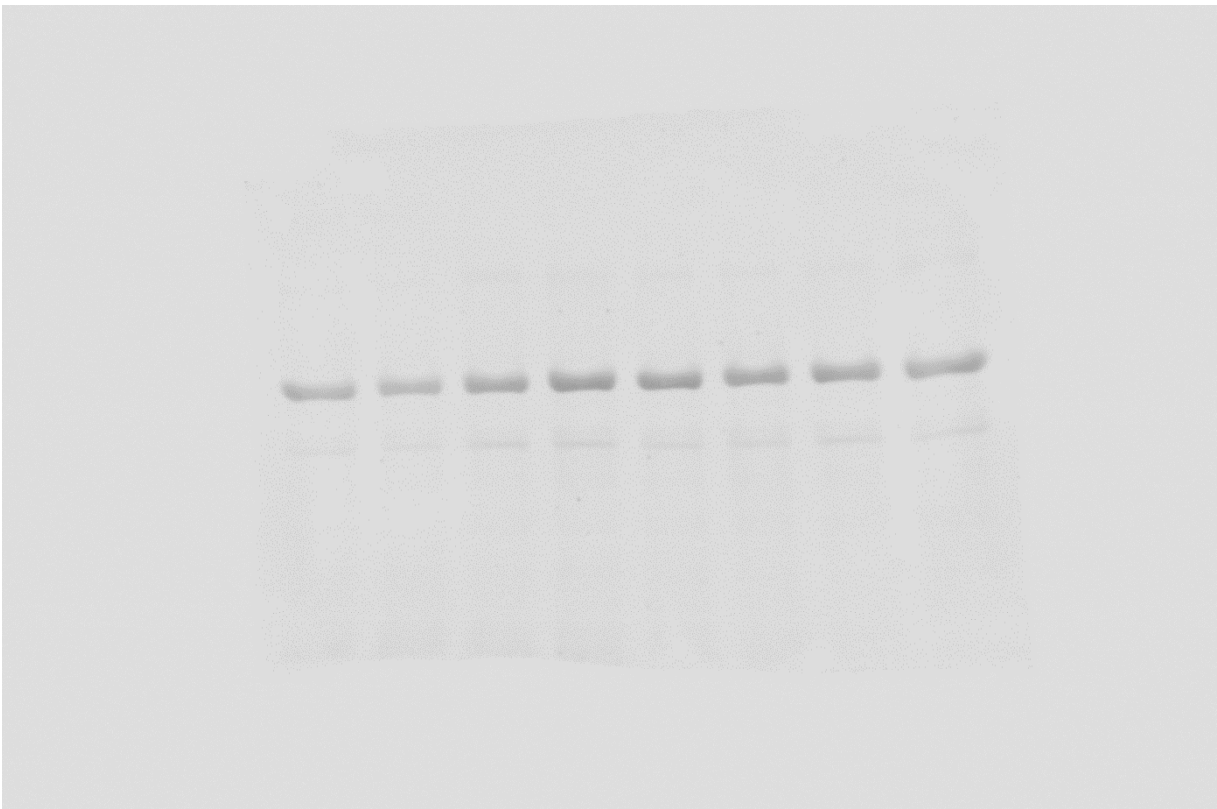

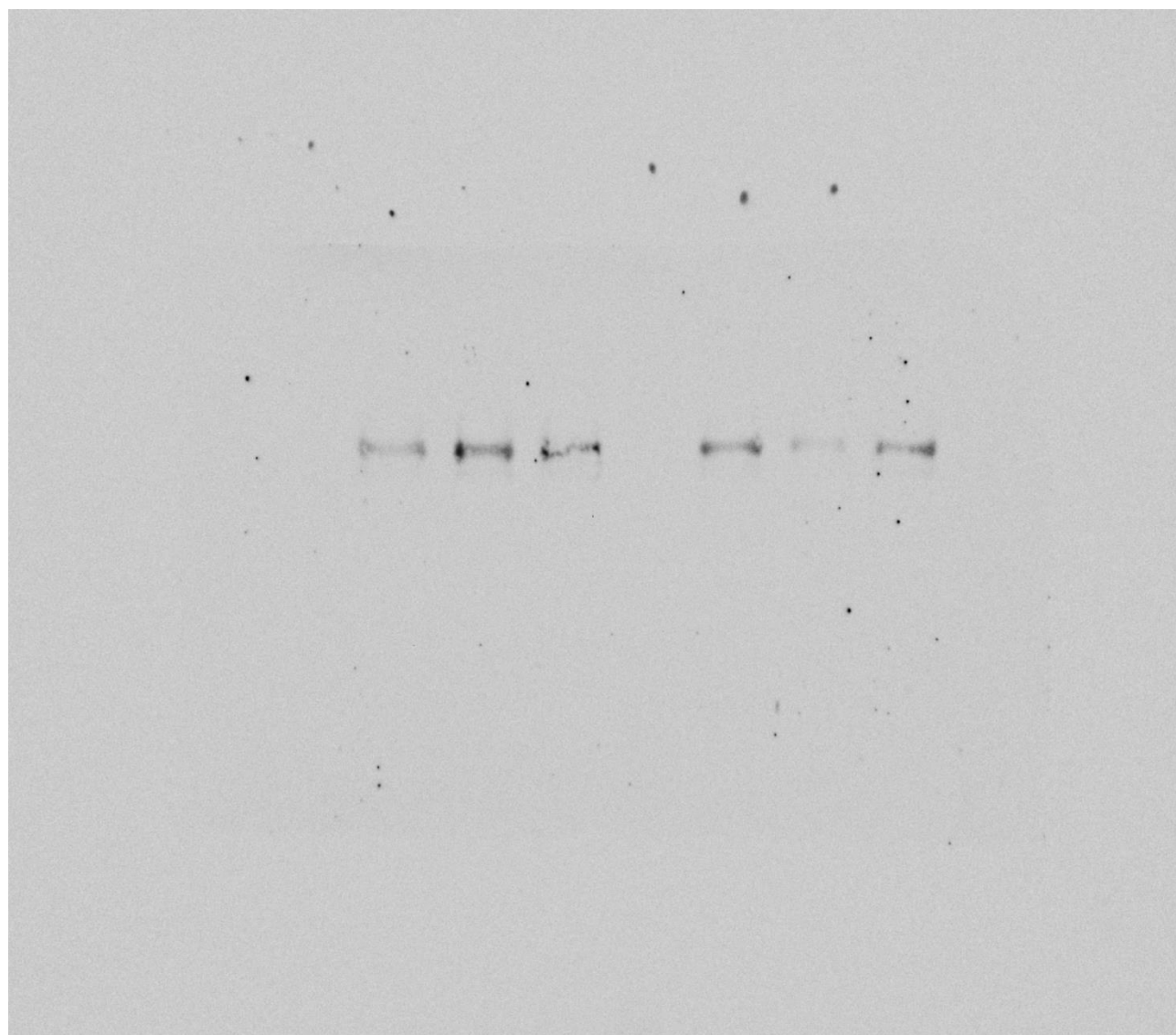

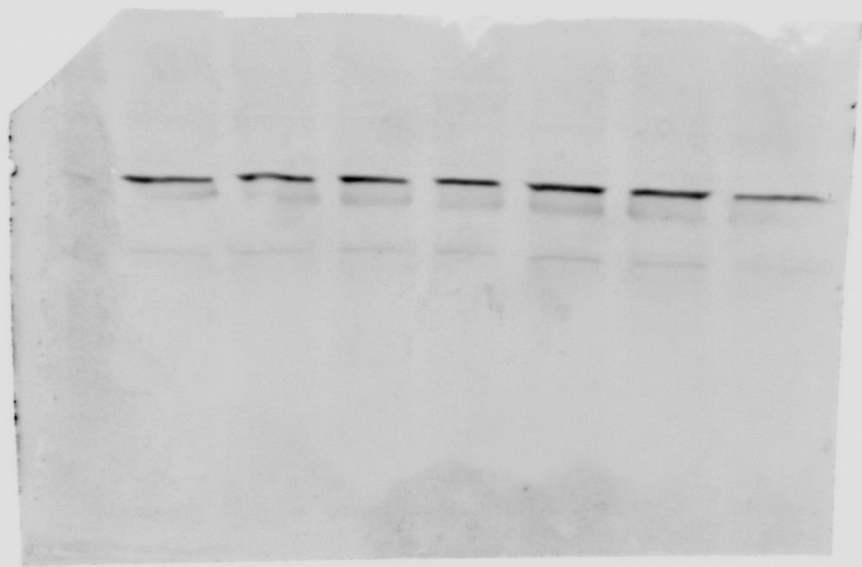

Supplement: Data Sheet 1 — Raw images of each representative western blot; images are displayed in the same order as in the main article. [file DataSheet_1.pdf]
